# Estimation of Coast-Wide Population Trends of Marbled Murrelets in Canada Using a Bayesian Hierarchical Model

**DOI:** 10.1371/journal.pone.0134891

**Published:** 2015-08-10

**Authors:** Douglas F. Bertram, Mark C. Drever, Murdoch K. McAllister, Bernard K. Schroeder, David J. Lindsay, Deborah A. Faust

**Affiliations:** 1 Environment Canada, Wildlife Research Division, Institute of Ocean Sciences, Sidney, British Columbia, Canada; 2 Environment Canada, Canadian Wildlife Service, Delta, British Columbia, Canada; 3 Fisheries Centre, University of British Columbia, Vancouver, British Columbia, Canada; 4 Bernard K. Schroeder Consulting, Nanaimo, British Columbia, Canada; 5 TimberWest Forest Corp., Nanaimo, British Columbia, Canada; 6 Kingston, Ontario, Canada; Estación Biológica de Doñana, CSIC, SPAIN

## Abstract

Species at risk with secretive breeding behaviours, low densities, and wide geographic range pose a significant challenge to conservation actions because population trends are difficult to detect. Such is the case with the Marbled Murrelet (*Brachyramphus marmoratus)*, a seabird listed as ‘Threatened’ by the *Species at Risk Act* in Canada largely due to the loss of its old growth forest nesting habitat. We report the first estimates of population trend of Marbled Murrelets in Canada derived from a monitoring program that uses marine radar to detect birds as they enter forest watersheds during 923 dawn surveys at 58 radar monitoring stations within the six Marbled Murrelet Conservation Regions on coastal British Columbia, Canada, 1996–2013. Temporal trends in radar counts were analyzed with a hierarchical Bayesian multivariate modeling approach that controlled for variation in tilt of the radar unit and day of year, included year-specific deviations from the overall trend (‘year effects’), and allowed for trends to be estimated at three spatial scales. A negative overall trend of -1.6%/yr (95% credibility interval: -3.2%, 0.01%) indicated moderate evidence for a coast-wide decline, although trends varied strongly among the six conservation regions. Negative annual trends were detected in East Vancouver Island (-9%/yr) and South Mainland Coast (-3%/yr) Conservation Regions. Over a quarter of the year effects were significantly different from zero, and the estimated standard deviation in common-shared year effects between sites within each region was about 50% per year. This large common-shared interannual variation in counts may have been caused by regional movements of birds related to changes in marine conditions that affect the availability of prey.

## Introduction

Population dynamics of widely-distributed species can vary strongly over their ranges [1, 2]. This variation can complicate the estimation and interpretation of temporal trends in abundance over time, especially when the underlying deterministic and stochastic mechanisms behind this variation remain unknown. This complication is especially problematic for species at risk, where management decisions and conservation actions often rely on assessment of temporal trends. Therefore, novel trend modeling approaches are needed that can simultaneously account for regional variation and provide estimates of population trends at a variety of scales. Such estimation is further complicated for species that exhibit secretive nesting behaviour, low densities during breeding and exhibit broad geographic ranges. These challenges exemplify the case of the Marbled Murrelet (*Brachyramphus marmoratus)*, a small marine bird of the Auk family which comes to land only to breed, and whose breeding range is virtually continuous from California to Alaska [3]. The species has been elusive and the first active nest in Canada was not discovered until 1993 [4] despite considerable historical conjecture and speculation [5]. Most marine birds nest in colonies which allow researchers to obtain detailed estimates of population size and vital rates. The Marbled Murrelet, however, does not form breeding colonies but instead exhibits secretive behaviour and nests solitarily at low densities (maximum of 0.090 +/- 0.060 birds per hectare [6]) in old growth trees up to 50 km from the ocean where the birds feed [7]. Marbled Murrelets have a slow life-history typical of seabirds, with age of first breeding at 2–5 years, low fecundity (0.17 to 0.22 female fledglings raised per female), high adult survival (0.83–0.92 per annum), and a generation time of approximately 10 years [8]. The secretive birds have cryptic colours and are generally quiet during breeding to avoid detection by predators. Marbled Murrelets do not construct a nest but lay a single egg on a large moss covered branch. When the egg hatches adults make daily provisioning trips, carrying fish crossways in their bill, to feed the nestling in the darkness of early morning, prior to dawn. Counting birds during these pre-dawn flights thus allows for links to be made between their abundance at sea and the availability of forest habitats.

The Marbled Murrelet is listed as Threatened, both in Canada under the *Species at Risk Act*, and in the United States under the *Endangered Species Act* for California, Oregon, and Washington states. Loss rates of old growth forest nesting habitats, rather than rigorous population trend estimates, are the main cause for species listings in both countries [9]. Threats from the marine environment include oil pollution and ship traffic, gill net mortality, and potential ocean climate change impacts on marine food webs [9]. The International Union for Conservation of Nature (IUCN) lists Marbled Murrelet as Endangered [10]. Despite being listed as a species at risk, the Marbled Murrelet is still a common bird in Canada, with a population crudely estimated to be approximately 99,000 birds [9,11], and therefore identifying appropriate management actions will also depend strongly on the rigorous assessment of temporal trends in abundance and interannual variation in counts.

The secretive breeding behaviour, low nesting density and large continuous breeding range of Marbled Murrelets complicate the establishment of a reliable way to estimate population sizes and to monitor trends over time. In the United States, Marbled Murrelet population size and trends have been estimated from annual replicate at sea-surveys over long areas of coastline from Washington State to California during the breeding season [3,12]. Marine radar has also been used to count Marbled Murrelets during breeding at strategic locations as they travel to and from watersheds where they nest. Research at five regions in British Columbia [6] and one in Washington state [13] showed a general linear relationship between the number of birds estimated with radar and the amount of old growth nesting habitat within Landscape Units. The general linear relationship has been used to set nesting habitat and population targets for the Canadian population in British Columbia [8,14,11]. To detect population trends, the Canadian Marbled Murrelet Recovery Team and collaborators concluded that radar was a more robust monitoring tool than at-sea surveys because the coefficients of variation (CV) for at-sea surveys were too high, and because replicate surveys would not be feasible for the vast extent of convoluted coastline of British Columbia. A radar monitoring design capable of detecting a decline of <1% per year was devised which called for 2–4 biannual surveys at 10–15 radar stations in each of British Columbia’s six conservation regions for 10–15 years. In 2006, Environment Canada implemented the coast-wide population trend detection program for the Marbled Murrelet in Canada based on the radar monitoring protocol.

In this paper, we couple the recent radar count estimates with available historical radar count datasets from the same sampling locations to construct the longest possible time series for the analyses of population trend to date in British Columbia, Canada, 1996 to 2013. We document the effects of important covariates, including tilt of radar device and day of year to account for within-season changes in abundance. As a seabird, the Marbled Murrelet shows annual fluctuations in abundance, likely related to how ocean foraging conditions affect breeding propensity and success [15,16,17], and therefore we developed a model that accounted for year effects shared across sites within a region to enhance the detection of long-term temporal trends. A Bayesian hierarchical modeling approach was taken in which the average count for individual sites and the slope of the underlying time trend, as well as year effects by region were treated as exchangeable random variables. This approach provided a statistically consistent conceptual framework to estimates trends in each site, within each region, and for the entire west coast within a single statistical model, and thus provides the most rigorous assessment of temporal trends for Marbled Murrelets within their range in Canada.

## Methods

### Study sites

**Fig 1:**
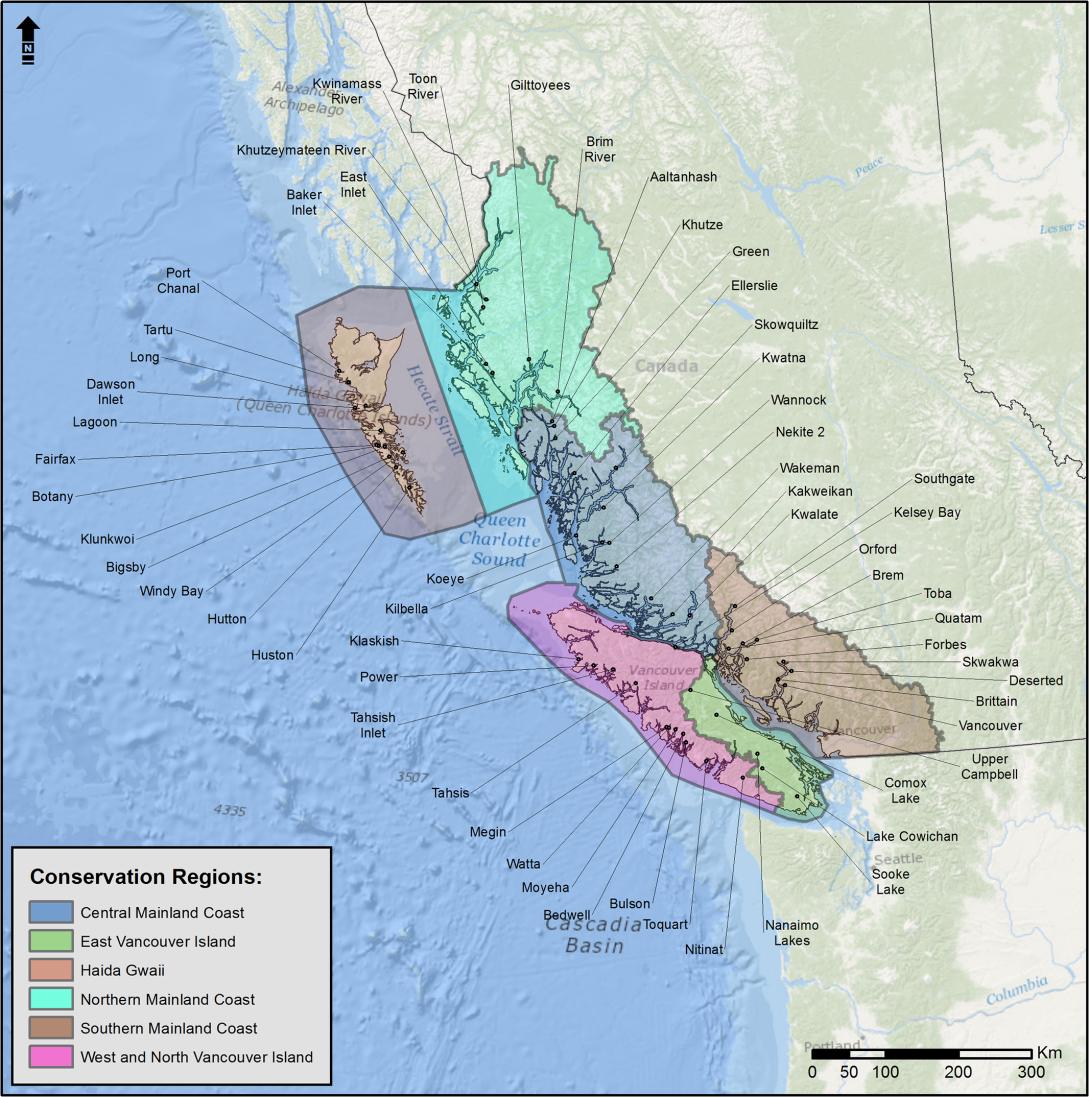
Environment Canada long-term radar monitoring stations in six Conservation Regions of British Columbia, as defined by the Canadian Marbled Murrelet Recovery Team [43].

The Canadian Marbled Murrelet Recovery Team divided British Columbia into six Marbled Murrelet Conservation Regions (Fig 1), and provided specific recovery targets for 2032 for each region [11,14]. To detect population trends, a stratified-sampling approach was used [18], and 10–11 survey stations per conservation region were identified (Fig 1) to be visited every 2–3 years, beginning in 2006. Within a year, each radar station was surveyed 3 times on non-consecutive days using standardized methods [19]. TimberWest Forest Corporation has conducted ongoing annual surveys at several sites on East Coast Vancouver Island (ECVI), 2003–2013. All of the ECVI sites are accessible by truck, in contrast to the other regions where most sites are only accessible by vessel and hence “boat-based” stations (except for Nitinat, Toquart, and Tahsis in the West and North Vancouver Island Conservation Region which can also be accessed by vehicle). We combined recent and historical data for selected long-term monitoring stations in British Columbia to test for trends in time within each region and for the entire coast of Western Canada. For logistical reasons, three stations that lie in the Central Mainland Coast were sampled in the same years as stations on the North Mainland Coast, and therefore had to be treated as part of North Mainland Coast Conservation Region to permit the calculation of Region-specific year effects (see *Trend Model*below). Surveys were conducted at radar stations in the hours before dawn, during which each target was identified as Murrelet by their size and speed, and tallied as in-bound or out-bound depending on its trajectory relative to the ocean. Weather conditions were recorded at the beginning and throughout the survey.

We obtained permission to work in Gwaii Haanas National Park Reserve and Haida Heritage Site from Parks Canada. We informed BC Parks that we were working in Port Chanal Ecological Reserve but did not require a permit because no one went ashore. Permission to work on private land on the East Coast of Vancouver Island was given by TimberWest Forest Corp. Permits were not required at other radar station locations (Fig 1). The Marbled Murrelet is listed as Threatened in Canada but permits were not required for the study because birds were not captured or handled.

### Abundance variables and data selection

Historical survey protocols included variable start times of 2 hours before sunrise and 90 min prior to sunrise, and some surveys were shorter than prescribed protocols due to obstruction of the radar signal by rain. Surveys which were used in these analyses were considered complete and did not have the significant screen obstruction from rain for more than 10 minutes continuously during the pre-sunrise period. Two measures of Marbled Murrelet abundance were considered: the maximum number of incoming or outgoing murrelets detected at each survey, and the number of incoming murrelets counted before dawn. The two measures were strongly correlated (ρ = 0.97, P = <0.001), and we therefore focused on the number of number of incoming murrelets counted before dawn, as this measure may best reflect the number of birds nesting in the upstream watersheds, made our study comparable to others [20] as well as minimizing possible double-counting of birds as they may make multiple trips from ocean foraging sites to nests.

### Covariates: Day of Year and Radar Tilt

Movements between nest sites and ocean foraging areas of Marbled Murrelets, and hence radar counts, vary widely within a single season from May to August, corresponding to the breeding chronology of egg-laying, incubation, and chick-rearing [21]. Further, this seasonal variation can differ strongly between Conservation Regions, with up to 30 days difference in peak activity between inner- and outer-coasts [22]. Day of the Year (DOY) when surveys were conducted ranged from 127 (May 7) to 227 (August 14), and we therefore included parameters to account for this within-season variation expected to occur in radar counts of Marbled Murrelets.

Detection rates of Marbled Murrelets is affected strongly by tilt of the radar unit [23], which had varied over time across a variety of values from 0.0 to 25.0 degrees within and among radar sites, and therefore we included a covariate to account for this effect in the time series of radar counts.

### Trend Model

We used a hierarchical Bayesian approach to account for the effects of covariates and to model temporal trends in radar counts at each site, which could then be consolidated to larger spatial scales of Conservation Region and coast-wide trend estimates. The model predicts radar counts of Marbled Murrelets, ${\hat{C}}_{s,y,i}$, such that the predicted count for observation *i* in year *y* at site *s* is given by:

$${\hat{C}}_{s,y,i}=\left(b_{o,s}+b_{1,s}\times (y_{s,i}-{\bar{y}}_{s})\right)\times \left(1+Y_{R,y}\right)\times (1+a_{R}{\left(D_{i}-c_{R}\right)}^{2})\times (1+t\times T_{i})$$ (1)

where *b*_*o*,*s*_ and *b*_*1*,*s*_ are the coefficients for the average radar count and year covariate *y*_*s*,*i*_ for observation *i* at site s, *Y*_*R*,*y*_ is the year effect for year *y* common to all sites in region *R*, *a*_*R*_ is the region-specific coefficient for the rate of change in detection from the mid-year point, *D*_*i*_ is the day of year for count *i* with 182 subtracted so that the covariate is centered at zero at the mid-point of the year, *c*_*R*_ is the region-specific coefficient for the deviation from the mid-year point where the detection rate is either maximum (or at a minimum, depending on the sign of *a*_*R*_) and *t* is the coefficient for the radar tilt covariate *T*_*i*_ associated with count *i*.

At the core of the estimation is the slope (*b*_*1*_) term that describes the average change in number of birds per year for a given site. The abundance intercept term (*b*_*o*_) for each site was referenced to the mean year for each site. Both *b*_*1*_ and *b*_*o*_ were treated as exchangeable across the 58 sites, and were distributed such that:

$$b_{1,s}∼\text{Normal}(\mu_{b1},\sigma_{b1}^{2})$$ (2A)

$$b_{o,s}∼\text{Normal}(\mu_{bo},\sigma_{bo}^{2})$$ (2B)

where the terms in the parentheses are the hyperparameters of the hierarchical model. This formulation was chosen after extensive exploratory modeling and sensitivity analysis, with dozens of different model implementations to test out different parameterizations related to effects of radar tilt, day of year, structural assumptions about year effects and hierarchical structure, and different settings for priors.

The inclusion of year effects allowed for the estimation of annual deviations away from the long-term trend that were shared among all sites in the same year within a Region, as could occur under variable regional ocean foraging conditions. For each Region, a reference year was chosen in which the year effect for this year was set to zero (*Y*_R,y = reference year_). The reference year was chosen as the central point in the time series for each region to minimize confounding with the estimation of the slope of the underlying temporal trend for each site. Since year effects were to reflect common-shared variation across all sites within a region, year effects were also fixed at zero for years that had few sites sampled within a region. Year effects for other years were estimated conditional on the set of years for which the year effect was fixed at zero. These estimated year effects had a prior distribution with a mean of zero, and standard deviation estimated by extending the hierarchical model form such that the prior density function for the standard deviation in year effects, *σ*_*Y*_, was given by:

$$\sigma_{Y}∼\text{LogNormal}\left(\mu_{Y},\sigma_{\sigma_{Y}}^{2}\right)$$ (3)

where the terms inside of the parentheses are the prior mean and prior variance in *σ*_*Y*_ which were both estimated. *σ*_*Y*_ was assumed to be the same for all regions. In Haida Gwaii where in 2003 there was only a single observation, it was not possible to estimate the year effect for 2003 and it was set to zero. The years 2004, 2005, and 2007 for Haida Gwaii also had very few observations and the year effects for these years were also fixed at zero. The year effects were fixed at zero for each of the remaining regions were: 2006 for East Vancouver Island, 2007 for West and North Vancouver Island, 1998, 2001, and 2009 for the North Mainland Coast, and 2000 and 2008 for the South Mainland Coast. Further, confounding would occur between year effect coefficients and the slope coefficient at sites with four or fewer years with observations; therefore year effects were set to zero for all sites on the Central Mainland Coast.

The prior probability distributions for the parameters in Eq 1 are described in Table 1. To allow the posterior distribution for parameters to be determined by the data, prior distributions were chosen to be vague in shape and with normal, uniform or lognormal form depending on whether the parameters were non-negative (S1 Table). The central tendency of the priors for some parameters that represented for example the standard deviation in some quantity, were chosen to be within an approximate order of magnitude of values typically obtained for these types of analyses. Some computer gaming with terms such as the date of year effect was also conducted prior to analyzing the data to ascertain plausible prior ranges of parameter values, e.g., for the a_R_ and c_R_ parameters. See Table 1 for further details on the prior probability distributions applied, and the code used in model specification is provided in S1 Text.

**Table 1:** Prior distributions for key parameters in trend model to predict radar counts for Marbled Murrelet in British Columbia, Canada. For the normal distribution, the 1^st^ parameter is the mean and the 2^nd^ parameter is the precision (1/ variance). For the gamma distribution, these are α and β. For the uniform these are the lower and upper bounds. For the lognormal the 1^st^ parameter is the natural logarithm of the median, and the 2^nd^ is the precision in the natural logarithm of the random variable.

**Parameter**	**Description**	**Distribution**	**1** ^**st**^ **param.**	**2** ^**nd**^ **param.**
a_R_	Day of year effect	Normal	0	10000
c_R_	Day of year effect	Normal	0	0.01
σ_R_	Residual error variance for each region R	Gamma	0.01	0.01
t	Radar tilt coefficient	Normal	0	1
σ_Y_	Hyper parameter for standard deviation of year effects	Log-normal	2.303	1.5625
*Y*_*R*,*y*_	Year effect	Normal	0	σ_Y_
*μ*_*bo*_	Hyper parameter for mean of intercepts	Uniform	1	2000
*σ*_*bo*_	Hyper parameter for standard deviation of intercepts	Log-normal	3.91	1.5625
*μ*_*b*1_	Hyper parameter for mean of slopes	Normal	0	0.0001
*σ*_*b*1_	Hyper parameter for standard deviation of slopes	Log-normal	2.30	1.5625
b_o,s_	Mean count for site	Log-normal	*μ*_*bo*_	*σ*_*bo*_
b_1,s_	Slope for site	Normal	*μ*_*b*1_	*σ*_*b*1_

### Likelihood functions of the radar count data

A lognormal density function was applied to model the probability density of each observed count (*C*) given the count $\hat{C}$ predicted by model parameters θ (ignoring subscripts site (s), year (y), observation within each site (i), except for region (R)):

$$P\left(C|\theta \right)=\frac{1}{C\sqrt{2\pi }\sigma_{R}}exp\left(-\frac{{\left(log\left(\frac{C}{\hat{C}}\right)\right)}^{2}}{2\sigma_{R}^{2}}\right)$$ (4)

The function accounted for the large variance in counts among sites and Conservation Regions, spanning two order of magnitudes, the strong positive skew in the distribution, and the low number of zero-value observations (n = 5), which were set to a value of one to allow use of the lognormal probability model of the data. Our model estimated a unique residual variance separately for each Conservation Region.

### Model Implementation and Convergence Diagnostics

We used program WinBUGS 1.4.3 (http://www.mrc-bsu.cam.ac.uk/bugs) to fit the hierarchical model, which provides a flexible platform for formulating models and conducting Monte Carlo Markov Chains (MCMC) analysis using Gibbs sampling. Two different chains were run in all instances, each with a different set of initial values. The Gelman-Rubin diagnostic statistic was computed for all slope and intercept terms and after 30,000 iterations indicated that the chains had stabilized about a stationary distribution. The burn-in was thus considered to be at 40,000 iterations. Posterior correlations for pairs of parameters all ranged between -0.6 and 0.6 and were mostly small in magnitude, i.e., mostly less than 0.4 in absolute value. Parameter values in the Markov Chains showed varying amounts of positive autocorrelation out to several dozen lag steps for some parameters. The models were however run for a sufficiently large number of MCMC iterations to enable sufficiently precise approximations of posterior statistics. The MC error in all instances for all statistics computed was less than about 3% of the computed posterior standard deviation after 80,000 additional iterations following the burn-in phase (i.e., the standard deviation in posterior means between theoretical MCMC runs would be about 3% of the true mean). Thus in all instances 80,000 was considered to be a sufficiently long run from which to obtain summary statistics. Thinning of the posterior was not appropriate since no model projections were carried out.

### Estimation of change in abundance

An estimate of relative trend was calculated as the slope/ intercept (= rate), where the intercept is referenced to the expected count in the mean year for each site, and thus provides an indication of proportional change over time. The rate was computed for each Conservation Region by taking the arithmetic average of the rate parameter values across sites within the Conservation Region. The rate was computed province-wide by taking the arithmetic average of the rate parameter across all sites in the province (n = 58). The probability of decline by site was computed as the proportion of runs within the burned-in portion of the iterations within the Monte Carlo Markov Chains in which the rate parameter for the site was negative. The same method was applied to compute the probability of decline by Conservation Region and province wide, based on the Region- and province-wide averages.

## Results

In total 170 parameters were estimated from 946 radar counts across 58 sites in 6 Conservation Regions from 1996 to 2013 (S1 Table). These parameters included 12 day of year (DOY) parameters, six standard error parameters, one tilt parameter, 30 year effect parameters, 58 slope parameters, 58 intercept parameters, and five hyperparameters (mean slope, mean intercept, SD in slope, SD in intercept, SD for year-effects). These hyperparameters captured the large temporal and spatial variation in radar counts over the entire British Columbia coast (Table 2). The posterior mean estimate of the SD in year effects was 0.48, indicating that common year effects could on average result in increase or decrease in counts away from the long-term trends by a factor of about 50% (Table 2). The 95% credibility interval of the posterior distribution for the hierarchical mean of the slope parameters between sites overlapped with zero, but had a strong update from the hyperprior distribution, suggesting that there was no overall negative or positive slope estimate across sites in the province (Table 2). The posterior distribution for the SD in slope estimates (average long-term changes in bird counts between years) suggested considerable variability among sites (Table 2). The posterior mean and SD for the intercept term, i.e., the average number of counts per site, suggested over an order of magnitude in variation among sites in the mean radar count (Table 2).

**Table 2:** Posterior medians and 95% credibility bounds for parameter estimates from hierarchical Bayesian model depicting temporal trends in the number of Marbled Murrelets counted at radar stations in coastal British Columbia, 1996–2013. Counts were based on the number of incoming Marbled Murrelets detected before dawn. Trends were based on radar surveys at 58 stations in 6 Conservation Regions (CC is Central Mainland Coast, EV is East Vancouver Island, WC is West and North Vancouver Island, HG is Haida Gwaii, NC is North Mainland Coast, and SC is South Mainland Coast). The day of year coefficient c was based on values with 182 subtracted from the Julian day to centre the values about the mid-year point. N = 943. Cases where 95% credibility interval does not overlap with zero are denoted in bold font.

**Parameter**	**Effect**	**Median**	**2.50%**	**97.50%**
T	Tilt effect	**0.096**	**0.061**	**0.147**
*σ*_*Y*_	SD of year effects: hyperparameter	**0.477**	**0.33**	**0.722**
*μ*_*b*1_	Mean slope: hyperparameter	-0.146	-1.298	1.113
*σ*_*b*1_	SD of slope: hyperparameter	**2.78**	**1.59**	**4.55**
*μ*_*bo*_	Mean intercept: hyperparameter	**64**	**48.1**	**84.1**
*σ*_*bo*_	SD of intercept: hyperparameter	**0.78**	**0.64**	**0.98**
a	Day of Year: a coefficient[HG]	**-5.38E-04**	**-6.51E-04**	**-3.94E-04**
a	Day of Year: a coefficient[NC]	-2.53E-04	-4.37E-04	1.41E-04
a	Day of Year: a coefficient[WC]	**-3.53E-04**	**-4.18E-04**	**-2.63E-04**
a	Day of Year: a coefficient[CC]	**1.11E-03**	**4.85E-04**	**2.20E-03**
a	Day of Year: a coefficient[EV]	**-2.63E-04**	**-3.84E-04**	**-8.87E-05**
a	Day of Year: a coefficient[SC]	**-3.13E-04**	**-5.99E-04**	**-1.17E-04**
c	Day of Year: c coefficient[HG]	**6.1**	**3**	**10.7**
c	Day of Year: c coefficient[NC]	-6.9	-20.5	11.7
c	Day of Year: c coefficient[WC]	**12.9**	**9.3**	**16.9**
c	Day of Year: c coefficient[CC]	**18.3**	**8.9**	**30**
c	Day of Year: c coefficient[EV]	1.8	-7.7	12.8
c	Day of Year: c coefficient[SC]	-2.3	-19.4	7.1

### Effects of Day of Year and Radar Tilt

**Fig 2:**
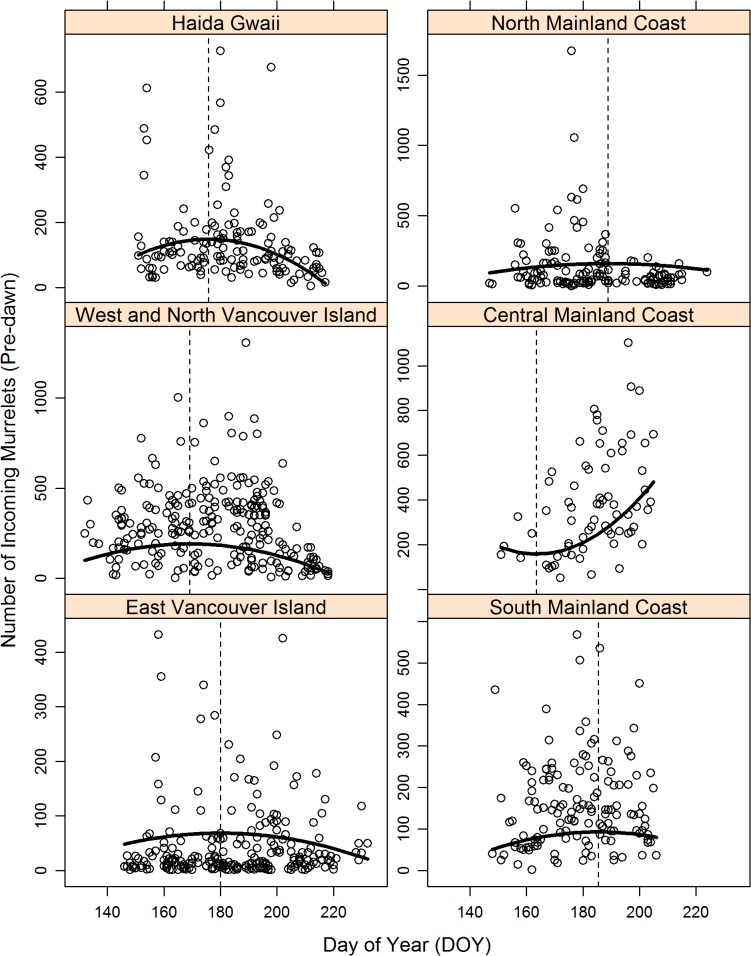
Variation in the number of Marbled Murrelets counted at radar stations in British Columbia, 1996–2013, as a function of Day of the Year (DOY), in six Marbled Murrelet Conservation Regions of British Columbia. Curved line for Day of Year plot shows quadratic relationship suggesting that, on average, highest counts are recorded between days 175–195 (June 18- July 8), with the exception of Central Mainland Coast, where peak counts occurred on July 24.

The day of year coefficients (Table 2) for five Conservation Regions (East Vancouver Island, West and North Vancouver Island, Haida Gwaii, North Mainland Coast, South Mainland Coast) indicated a non-linear seasonal pattern in radar counts, with increases followed by decreases and maximum counts occurring near days of year 169 to 189 (18 June to 8 July, on non-leap years; Fig 2). Seasonal patterns appeared different in the Central Mainland Coast, where a-coefficient was significant and positive, suggesting that counts tended to increase with time away from date of minimum count that occurred on day of year 164 (June 13, on non-leap years).

The posterior estimates for the tilt coefficient was significant and positive, indicating that more murrelets were counted when the radar unit was tilted upwards: for example, for a tilt of 12.3 degrees gives a posterior mean multiplicative effect of about 2.2 (Table 2). However, high positive autocorrelation existed in the Markov Chains for the tilt coefficient, suggesting that there may be some confounding between tilt effect and other parameters, e.g., the intercept or year effects, for some areas where radar tilt values were mostly high or low.

### Trends in murrelet counts over time

**Fig 3:**
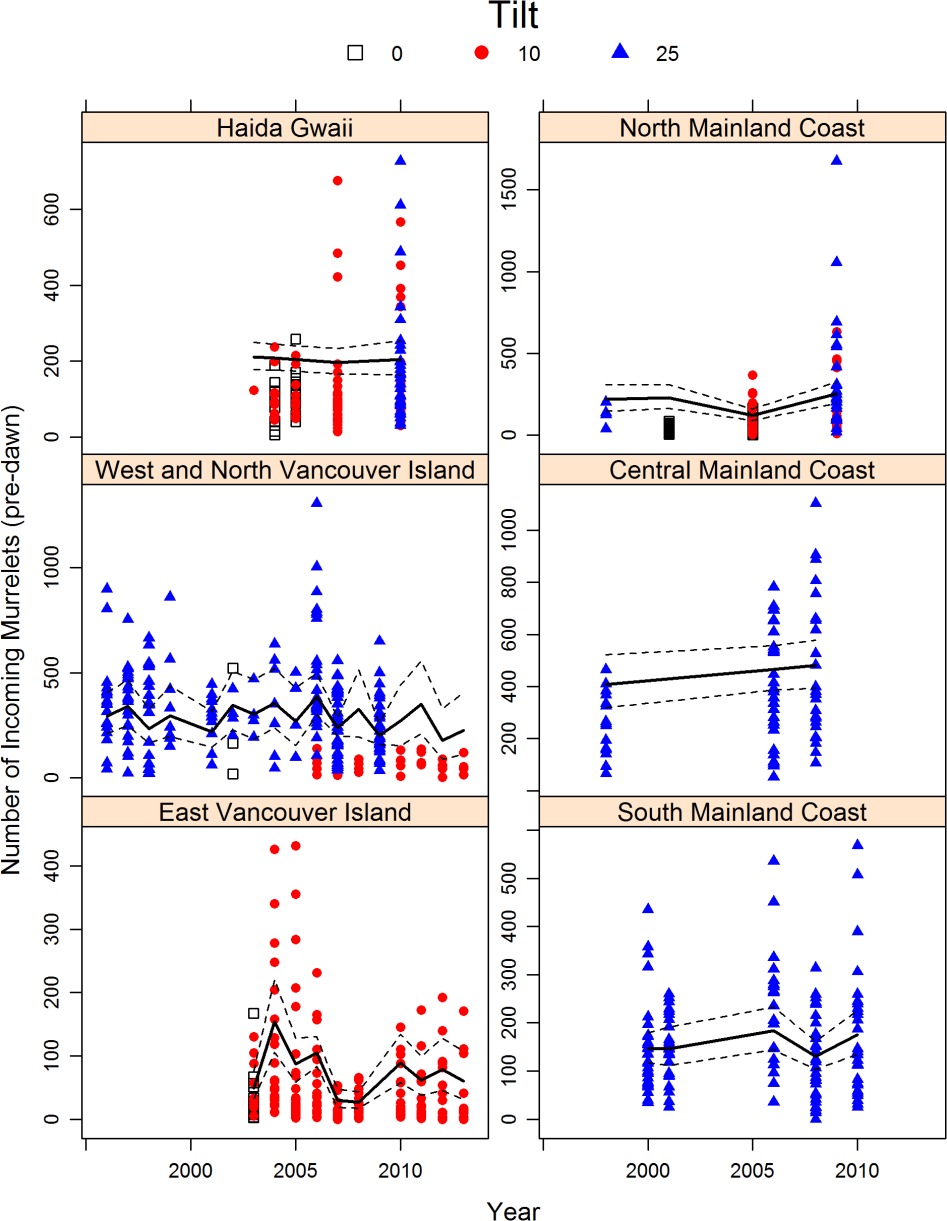
Temporal trends in the number of Marbled Murrelets counted at radar stations distributed through six Conservation Regions in British Columbia, 1996–2013. Thick lines indicate predicted means from trend model, with 95% credibility intervals that depict the within-Region yearly-variation. Colour and shape of symbol indicate radar tilt: 0 (which include 0 and 5.2 degree tilts, open squares), 10 (which include 10, 11.3 and 12.8 degrees, red circles) and 25 degrees (blue triangles).

The rate of change (slope/intercept) at each site provided a measure of potential long-term trends in abundance, expressed as proportional rate of change, with common year effects within a region removed. The mean rate of change province-wide was negative, with a posterior median of -1.6%, with a 95% credibility interval of -3.2%/yr to 0.01%/yr that included 0, but a 90% credibility interval that did not, indicating moderate evidence for a temporal decline in counts over the province as a whole (Table 3). Considerable variation existed among sites in their rates of change of counts over time, however, and varied from a minimum of -21%/yr (Nanaimo Lakes, East Vancouver Island) to a maximum of 8%/yr (Klunkwoi, Haida Gwaii). Of the 58 monitored sites, 9 sites had rate of change estimates had 95% credibility intervals that did not overlap 0: Lake Cowichan, Nanaimo Lakes, and Sooke Lake on East Vancouver Island; Fairfax and Klunkwoi on Haida Gwaii; and Brittain, Deserted and Southgate in the South Mainland Coast (Table 3). These estimates of rate of change were negative for all of these areas except for Klunkwoi. At the 90% level, an additional four sites had rates of change credibility limits that did not encompass 0, including Comox Lake on East Vancouver Island, Hutton on Haida Gwaii, Watta on West and North Vancouver Island, and Skowquiltz on Central Mainland Coast. When aggregated to Conservation Regions, the mean rates of change for East Vancouver Island (median value -8.6%) and South Mainland Coast (median -3.1%) had 95% credibility intervals that did not encompass 0, but not for the other Regions (Fig 3). At the 90% level, the rate of change at Haida Gwaii Region also had negative trend (median: -3.3%) with credibility limits that did not encompass 0.

**Table 3:** Posterior medians, 95% credibility limits, and 90% credibility limits for temporal trends in radar counts of Marbled Murrelets at 58 sites in six Conservation Regions in British Columbia, 1996–2013. P(decline) is the probability of decline, computed as the proportion of model runs in which the rate parameter for the site was negative (see Methods). Regions are in italics. Cases where the 95% or 90% credibility interval did not overlap with zero are designated in bold font.

**Region/Site**	**Median**	**Lower 95% CL**	**Upper 95% CL**	**Lower 90% CL**	**Upper 90% CL**	**P(decline)**
Coast-wide	**-0.016**	-0.032	0.0001	**-0.030**	**-0.003**	0.97
*Haida Gwaii*	**-0.033**	-0.074	0.002	**-0.067**	**-0.004**	0.97
Bigsby	-0.033	-0.121	0.038	-0.106	0.028	0.81
Botany	-0.067	-0.172	0.031	-0.156	0.016	0.9
Dawson Inlet	0.024	-0.066	0.12	-0.052	0.104	0.3
Fairfax	**-0.141**	**-0.275**	**-0.014**	**-0.255**	**-0.031**	0.99
Huston	**-0.118**	**-0.2**	**-0.019**	**-0.189**	**-0.035**	0.99
Hutton	-0.085	-0.189	0.014	**-0.172**	**-0.001**	0.95
Klunkwoi	**0.082**	**0.007**	**0.149**	**0.019**	**0.139**	0.02
Lagoon	0	-0.09	0.082	-0.075	0.069	0.5
Long	-0.032	-0.102	0.026	-0.091	0.017	0.85
Port Chanal	0.003	-0.024	0.033	-0.019	0.028	0.4
Tartu	-0.02	-0.107	0.053	-0.091	0.041	0.71
Windy Bay	-0.008	-0.075	0.049	-0.061	0.039	0.61
*North Mainland Coast*	0.014	-0.013	0.043	-0.009	0.038	0.16
Aaltanhash	0.022	-0.078	0.105	-0.063	0.096	0.33
Brim River	0.01	-0.074	0.088	-0.061	0.076	0.41
East Inlet	0	-0.082	0.085	-0.068	0.070	0.5
Gilttoyees	0.007	-0.074	0.086	-0.061	0.073	0.43
Green	-0.011	-0.083	0.055	-0.071	0.044	0.63
Khutze	0.046	-0.019	0.098	-0.008	0.091	0.08
Khutzeymateen River	0.036	-0.034	0.116	-0.023	0.104	0.17
Kwinamass River	0.019	-0.017	0.082	-0.012	0.069	0.16
Toon River	-0.001	-0.051	0.048	-0.041	0.039	0.52
*West and North Vancouver Island*	0	-0.021	0.022	-0.017	0.018	0.49
Bedwell	-0.016	-0.054	0.025	-0.048	0.018	0.78
Bulson	-0.017	-0.053	0.026	-0.048	0.019	0.79
Kelsey Bay	0.035	-0.054	0.099	-0.039	0.091	0.21
Klaskish	-0.015	-0.072	0.033	-0.062	0.025	0.73
Megin	-0.02	-0.067	0.023	-0.059	0.016	0.81
Moyeha	0.012	-0.022	0.052	-0.017	0.046	0.25
Nitinat	-0.031	-0.122	0.044	-0.106	0.032	0.78
Power	-0.002	-0.051	0.05	-0.042	0.041	0.53
Tahsis	-0.003	-0.073	0.072	-0.063	0.060	0.53
Tahsish	0	-0.039	0.039	-0.032	0.032	0.51
Toquart	0.004	-0.048	0.065	-0.039	0.053	0.43
Watta	**0.045**	-0.002	0.086	**0.006**	**0.080**	0.03
*Central Mainland Coast*	0.015	-0.008	0.037	-0.005	0.034	0.1
Kwalate	-0.02	-0.102	0.051	-0.088	0.041	0.7
Kakweikan	0.031	-0.02	0.077	-0.012	0.071	0.12
Wakeman	-0.002	-0.07	0.058	-0.058	0.049	0.52
Wannock	0.008	-0.054	0.059	-0.043	0.052	0.39
Kilbella	-0.006	-0.055	0.035	-0.046	0.029	0.62
Koeye	0.039	-0.024	0.089	-0.014	0.082	0.11
Kwatna	0.015	-0.021	0.055	-0.015	0.049	0.21
Skowquiltz	**0.066**	-0.013	0.11	**0.001**	**0.105**	0.05
Ellerslie	0.043	-0.008	0.093	-0.001	0.086	0.05
Nekite	-0.013	-0.129	0.092	-0.107	0.073	0.6
*East Vancouver Island*	**-0.086**	**-0.126**	**-0.028**	**-0.120**	**-0.039**	1
Lake Cowichan	**-0.102**	**-0.145**	**-0.006**	**-0.141**	**-0.026**	0.98
Upper Campbell	0.031	-0.046	0.114	-0.035	0.101	0.22
Comox Lake	**-0.077**	-0.132	0.009	**-0.125**	**-0.006**	0.96
Nanaimo Lakes	**-0.207**	**-0.285**	**-0.105**	**-0.272**	**-0.122**	1
Sooke Lake	**-0.155**	**-0.231**	**-0.023**	**-0.219**	**-0.047**	0.99
*South Mainland Coast*	**-0.031**	**-0.058**	**-0.005**	**-0.053**	**-0.009**	0.99
Brem	-0.003	-0.052	0.046	-0.044	0.038	0.55
Brittain	**-0.103**	**-0.145**	**-0.043**	**-0.139**	**-0.053**	1
Deserted	**-0.092**	**-0.169**	**-0.002**	**-0.158**	**-0.016**	0.98
Forbes	-0.046	-0.097	0.019	-0.090	0.008	0.92
Orford	0.022	-0.038	0.079	-0.028	0.070	0.24
Quatam	-0.036	-0.097	0.028	-0.088	0.018	0.86
Skwawka	0.044	-0.023	0.095	-0.011	0.088	0.09
**Southgate**	**-0.09**	**-0.159**	**-0.006**	**-0.149**	**-0.020**	0.98
Toba	-0.002	-0.054	0.055	-0.046	0.045	0.52
Vancouver	-0.016	-0.085	0.055	-0.075	0.044	0.66

### Year effects

For the five regions in which common year effects were estimated, significant year effects were found in East Vancouver Island, West and North Vancouver Island, North Mainland Coast, and South Mainland Coast Conservation Regions but not for Haida Gwaii (Table 4). The estimates for the standard deviation (SD) term in the lognormal likelihood functions varied among the 6 Conservation Regions, with the lowest SD for the South Mainland Coast (mean of 0.42, SD of 0.04) and the highest for East Vancouver Island (mean of 0.93, SD of 0.05).

**Table 4:** Posterior medians and 95% bounds for year-effect coefficients in radar counts of Marbled Murrelets at six Conservation Regions in British Columbia, 1996–2013. CC is Central Mainland Coast, EV is East Vancouver Island, WC is West and North Vancouver Island, HG is Haida Gwaii, NC is North Mainland Coast, and SC is South Mainland Coast. Cases where the 95% credibility interval does not overlap with zero are designated in bold font.

**Region**	**Year**	**2.50%**	**Median**	**97.50%**
HG	2010	-0.163	0.097	0.445
**NC**	**2005**	**-0.626**	**-0.502**	**-0.333**
WC	1996	-0.154	0.216	0.758
**WC**	**1997**	**0.010**	**0.422**	**0.992**
WC	1998	-0.321	-0.027	0.394
WC	1999	-0.198	0.236	0.871
WC	2001	-0.407	-0.090	0.373
WC	2002	-0.056	0.448	1.169
WC	2003	-0.233	0.270	0.986
WC	2004	-0.017	0.494	1.206
WC	2005	-0.355	0.128	0.817
**WC**	**2006**	**0.250**	**0.627**	**1.127**
WC	2008	-0.163	0.382	1.153
WC	2009	-0.360	-0.143	0.147
WC	2010	-0.329	0.150	0.845
WC	2011	-0.090	0.495	1.319
WC	2012	-0.613	-0.244	0.373
WC	2013	-0.512	-0.037	0.679
**EV**	**2003**	**-0.736**	**-0.564**	**-0.278**
EV	2004	-0.084	0.406	1.044
EV	2005	-0.481	-0.188	0.227
**EV**	**2007**	**-0.818**	**-0.704**	**-0.522**
**EV**	**2008**	**-0.832**	**-0.724**	**-0.549**
EV	2009	-0.411	-0.028	0.487
EV	2010	-0.615	-0.303	0.162
EV	2011	-0.533	-0.078	0.575
EV	2012	-0.678	-0.263	0.408
SC	2001	-0.233	0.014	0.332
**SC**	**2006**	**0.034**	**0.370**	**0.798**
**SC**	**2010**	**0.051**	**0.392**	**0.836**

## Discussion

Our trend model for radar counts of Marbled Murrelets attributed hierarchical structure to the values for slope and intercept at each site, such that trends in counts at each site could be averaged upwards to Conservation Regions and coast-wide. The model estimated covariates for tilt and seasonal variation (day of year effects, estimated separately by Conservation Region), a limited set of year effects, and residual variation separately by Conservation Region. Therefore, this model accounted for variable radar tilt over time, allowed for Region-wide fluctuations away from the long-term trend as could occur from variable ocean foraging conditions experienced among different regions and years, and was applied to the largest data set of radar counts of Murrelets available for every Conservation Region. This model indicated, on average, radar counts of incoming Marbled Murrelets on the BC coast declined over time at rate of -1.6%/year from 1996 to 2013 with a 95% credibility interval that overlapped with 0, but with a 90% credibility interval which did not. This decline however varied widely among Conservation Regions and radar sites, with strongest declines occurring at East Vancouver Island (-8.6%/yr) and South Mainland Coast (-3.1%/yr), and likely at Haida Gwaii (-3.3%/yr, significant based on the 90% credibility interval). Based on the lower bound of the credibility interval for the province-wide trend, we estimate that sufficient statistical power was present to detect a province-wide trend of absolute value of approximately >4%, based on a 95% credibility interval, given the strong inter-regional variation in trends, the residual error variance, and the number of years and sites in the current data set.

The strong variation in trends among sites and Regions may be compared to help identify potential causal mechanisms behind population changes in murrelets. A thorough examination of the causes for population trends is well beyond the scope of this study, but to aid in the interpretation of our results and to improve the design of future work, it is important to briefly address key factors that could influence the trends. The contribution of reproductive success to long-term trends in abundance of Marbled Murrelets on the Pacific Coast is influenced by the combined effects of availability of suitable forest nesting habitat and the interannual variation in marine prey, both of which can vary widely among the six Conservation Regions. The Regions that had negative trends, East Vancouver Island and the South Mainland Coast, had some of the largest reductions in forest nesting habitat that occurred between 1978 and 2008 [24]. However, these trends in southern Regions may have also resulted in changes in the marine environment, as availability of marine prey is positively associated with reproductive success of murrelets [15,16,25,26,27,28]. A number of other piscivorous bird species have also declined in the Strait of Georgia between 1990 and 2010 [29,30,31,32], the body of water where these murrelets are most likely feeding, indicating the potential for a marine community-level effect in addition to changes that may have occurred in the murrelets’ terrestrial habitat. Rigorous studies of changes in marine prey availability and their impacts on Marbled Murrelets are fundamental to our ability to attribute causes of population trends but are currently lacking.

Radar counts are positively related to amounts of old growth nesting habitats [6,13], so it is generally expected that changes in bird numbers heading inland would be similar to changes in amounts of nesting habitats. This association occurred at the broad spatial scale of Conservation Regions, with negative trends on the East Coast Vancouver Island and on the South Mainland Coast, where reductions in nesting habitat occurred between 1978 and 2008 [24]. Positive trends in radar counts were detected in the North Mainland Coast and Central Coast Conservation Regions, where reductions in nesting habitat have been less acute, or where habitat may have increased depending on the criteria used to define nesting habitat [24]. These differences support the hypothesis that murrelets may be limited by availability of forest nesting habitat, although the small sample size (n = 6 Regions) and poor overlap in time (1978–2008 versus 1996–2013) precludes rigorous assessment of correlations between trends in radar counts and rates of forest habitat change at the regional spatial scale. However, given advances in mapping of nesting habitat of Marbled Murrelets in British Columbia [14], potential exists for a more rigorous assessment. Further, the strong variation that we observed at the station level indicates that it will be instructive to analyze variation in radar count data in relation to forest conditions due to historical and present forestry practises [33], hydro, mining practices, and land status (e.g., private ownership, provincial, protected area) at the watershed-unit level. Such an analysis would be facilitated by the current hierarchical Bayesian modeling approach, which estimates trends at individual sites that are averaged upward to larger spatial scales (Conservation Region and province-wide in this case) as derived parameters. This approach provides flexibility to average over other spatial arrangements (e.g., oceanographic domains, or forestry management units) and thus may be used for inference about specific mechanisms underlying temporal trends.

We found that the inclusion of year effects in the model provided a useful separation of the annual Region-wide fluctuations from the underlying long-term trend, and provided some protection against long-term trends being affected by the specific conditions that might have occurred at the start of each time series. Given the low reproductive capacity of Marbled Murrelets [8], and that available evidence suggests that Marbled Murrelets show variable breeding site fidelity [34], such Region-wide fluctuations are likely associated with yearly differences in ocean conditions that impact prey availability and which result in dispersal in and out of the Conservation Regions. It is therefore important to note that movements of breeding birds between Conservation Regions in response to local breeding failures could also affect estimates of population trends.

Ocean conditions show strong year-specific variation over large spatial scales on the British Columbia coast [35]. In 1998, marine prey populations over much of southern British Columbia were greatly reduced during the large scale El Niño Southern Oscillation event [36]. In 2005, an unusual atmospheric blocking event severely delayed spring transition winds and hence marine production in the California Current Ecosystem [37]. Colonial seabirds off the West Coast of Washington [38] and Vancouver Island [39, 40] failed to breed in those two years. Thus, concurrent movements of marbled murrelets away from marine areas that lack prey could explain the interannual variation in radar counts. In 2005, Marbled Murrelet researchers on the West Coast of Vancouver Island noted large reductions in the number of birds observed on the water and poor recruitment in the south at Carmanah Light Station [16]. At 10 radar stations on the north end of Vancouver Island, 68% fewer murrelets were counted in 2005 compared to 2004 ([41], J Deal, Western Forest Products Ltd, *personal communication*). During those same years, radar counts at watersheds on the west coast in Haida Gwaii were 14% higher in 2005 than in 2004 with the largest increases (16–130%, average 65%) confined to watersheds on the west coast of the archipelago [42]. The large increases in abundance observed in Haida Gwaii are consistent with a northward movement of birds from Vancouver Island in 2005 when ocean feeding conditions in southern British Columbia were poor. In Washington State, annual replicate at-sea surveys of Marbled Murrelets during 2005 in several distinct sampling zones also showed significant movements of birds out of Juan de Fuca Strait (adjacent to southern-western Vancouver Island) and into the San Juan Islands where they do not breed [17]. Therefore, the inclusion a year-specific term that varies by Conservation Region has a strong biological rationale.

A significant decline of -9%/yr in radar counts of Marbled Murrelets was detected on the East Vancouver Island Conservation Region between 2003 and 2013. This Region had the largest residual variance, and it is plausible that radar counts in this Region are more strongly affected by movements of birds than in other Regions. The high counts early in 2005 may have resulted from birds with failed nesting attempts from outside the Region moving through the radar stations on the east side of the Vancouver Island. The entire East Coast Vancouver Island Conservation region has approximately 1000–2000 birds [9], so it is very unlikely that, for example, the record high 400+ birds observed at Comox Lake in 2005 were all local breeders. Further, radar stations on Vancouver Island are inland, often associated with open water lakes, such that bird movement would be less constrained than at coastal radar stations in other conservation regions where flight paths are more generally constrained by the topography of steep walled fjords. The population on East Vancouver Island is small and adjacent to regions with much larger populations (West Coast and North Vancouver Island, South Mainland Coast, and Puget Sound/Strait of Juan de Fuca region of Washington State with 4,393 birds [17], and we note that trends on the East Coast of Vancouver Island may be much more sensitive to influence from interannual movements of comparatively large numbers of birds from other regions. We tested for this contingency by comparing year effects from East Vancouver Island to the adjacent West Coast and North Vancouver Island, and only found a weak negative correlation (ρ = -0.15, n = 9), providing only weak evidence for movements between these two Conservation Regions. Nonetheless, quantification via tracking of bird movements during the breeding season will help to identify the mechanisms behind population changes at this Region, and allow separation of marine influences on reproduction from changes in the amount and quality of terrestrial nesting habitats.

Our results based on radar counts are consistent with Marbled Murrelet population trends derived from at-sea counts in British Columbia and elsewhere in the species’ breeding range in North America for similar time periods. At-sea counts in Pacific Rim National Park Reserve, which lies within the West Coast and North Vancouver Island Conservation Region, indicate Marbled Murrelets counts declined from 1996 to 2011 (-3.1%/yr) (Zharikov et al. 2012 in [40],[3],[8]). However, this negative trend has been diminishing due to an increase from 2006, with record high years in 2010 and 2011 [40], which is consistent with the stable trend we observed in this Region. In Laskeek Bay in Haida Gwaii, a time series of at-sea counts from 1991 to 2009 had a strong negative trend of -11%/yr, which plateaus for the more recent portion of the time series from 1999 to 2009 [9], which is consistent with the weakly negative trend in radar counts from the Haida Gwaii Conservation Region. This consistency indicates that at-sea counts and radar dawn surveys are useful complements in overall monitoring efforts of murrelet populations in British Columbia.

Elsewhere in the species’ breeding range in North America, long-term monitoring of Marbled Murrelets in US Pacific Northwest (Washington, Oregon, and California) from 2001 to 2013 indicated a similar pattern that we observed in British Columbia, a weak overall decline for the overall area (-1.2 percent per year; 95% confidence interval: -1.2 to 0.5), with strong regional differences in the rates of change [12,44]. In particular, counts of murrelets at the two Conservation Zones in Washington, adjacent to British Columbia, declined at similar rates that we observed (Conservation Zone 1 [-3.9 percent decline per year; 95% confidence interval: -7.6 to 0.0], which includes the Strait of Juan de Fuca, San Juan Islands, and Puget Sound; and Conservation Zone 2 [-6.8 percent decline per year; 95% confidence interval: -11.4 to -1.9], which includes the outer coast of Washington). Therefore, declines in southern British Columbia likely form part of a larger regional decline in murrelet populations related to reductions in forest nesting habitat and changes in prey fish populations [12,44].

North of British Columbia, long-term monitoring of *Brachyramphus* murrelet populations in Prince William Sound, Alaska, has also shown declines at a rate of approximately -2%/year, as part of a continued declined since the 1970s attributed to changes in the marine environment, given that the amount of past and current timber harvest in the area has been comparatively minor [45,46]. We found little evidence of declines in the northern and central British Columbia, indicating the diversity of population drivers that may occur through the species’ breeding range.

The Canadian population of Marbled Murrelets has been estimated to be 99,100 (72,600–125,600) birds, with 8,000–25,000 on Haida Gwaii, 18,400–26000 on the Northern Mainland Coast, 20,000–42,000 on the in Central Mainland Coast, 6,000–7,000 on the South Mainland Coast, 18,700–23,600 on the West and North Vancouver Island and 1000–2000 on East Vancouver Island [9, 11]. It is worth noting that the declines have occurred at Conservation Regions with substantially fewer birds, although this difference likely did not strongly affect the estimate of province-wide trend. Each site received approximately equal weight in the province-wide estimate of rate of change in abundance, and given that the Conservation Region with the lowest estimated abundance also had the lowest number of sites, this Region had reduced weight in the estimate of province-wide decline. Improving the rigour of the population size estimates and integrating them with estimates of temporal trends will provide a better assessment of status of murrelet populations in the province as a whole, and help to improve the overall context in which conservation decisions are made in Canada.

## Supporting Information

S1 Table Parameter values for a hierarchical Bayesian model estimating temporal trends in Marbled Murrelets from counts using marine radar deployed before dawn at 58 stations in 6 Conservation Regions along the coast of British Columbia. Two letter codes refer to conservation regions (Fig 1): CC is Central Mainland Coast, EV is East Vancouver Island, WC is West and North Vancouver Island, HG is Haida Gwaii, NC is North Mainland Coast, and SC is South Mainland Coast.
(DOCX)

S2 Table Names and locations for radar monitoring stations for Marbled Murrelets at 6 Conservation Regions on the coast of British Columbia, Canada. (DOCX)

S3 Table Radar counts of Marbled Murrelets at six Conservation Regions on the coast of British Columbia, Canada. (XLSX)

S1 Text WinBugs code for hierarchical regression model predicting pre-dawn radar counts as function of year, seasonal effects (Day of Year; DOY), and radar tilt. (DOCX)

S1 Fig Individual radar station time series data at Marbled Murrelet Conservation Region Haida Gwaii. Symbol indicates radar tilt: 0 (which include 0 and 5.2 degree tilts, open squares), 10 (which include 10, 11.3 and 12.8 degrees, red circles) and 25 degrees (blue triangles). Lines are yearly estimates for each Region, as predicted by the trend model along with 95% credibility intervals.
(TIF)

S2 Fig Individual radar station time series data at Marbled Murrelet Conservation Region North Mainland Coast. Symbol indicates radar tilt: 0 (which include 0 and 5.2 degree tilts, open squares), 10 (which include 10, 11.3 and 12.8 degrees, red circles) and 25 degrees (blue triangles). Lines are yearly estimates predicted by the trend model, along with 95% credibility intervals.
(TIF)

S3 Fig Individual radar station time series data at Marbled Murrelet Conservation Region Central Mainland Coast. Symbol indicates radar tilt: 0 (which include 0 and 5.2 degree tilts, open squares), 10 (which include 10, 11.3 and 12.8 degrees, red circles) and 25 degrees (blue triangles). Lines are yearly estimates predicted by the trend model, along with 95% credibility intervals.
(TIF)

S4 Fig Individual radar station time series data at Marbled Murrelet Conservation Region South Mainland Coast. Symbol indicates radar tilt: 0 (which include 0 and 5.2 degree tilts, open squares), 10 (which include 10, 11.3 and 12.8 degrees, red circles) and 25 degrees (blue triangles). Lines are yearly estimates predicted by the trend model, along with 95% credibility intervals.
(TIF)

S5 Fig Individual radar station time series data at Marbled Murrelet Conservation Region West and North Vancouver Island. Symbol indicates radar tilt: 0 (which include 0 and 5.2 degree tilts, open squares), 10 (which include 10, 11.3 and 12.8 degrees, red circles) and 25 degrees (blue triangles). Lines are yearly estimates predicted by the trend model, along with 95% credibility intervals.
(TIF)

S6 Fig Individual radar station time series data at Marbled Murrelet Conservation Region East Coast Vancouver Island. Symbol indicates radar tilt: 0 (which include 0 and 5.2 degree tilts, open squares), 10 (which include 10, 11.3 and 12.8 degrees, red circles) and 25 degrees (blue triangles). Lines are yearly estimates predicted by the trend model, along with 95% credibility intervals.
(TIF)
