# Supplementary material for: Estimation of Coast-Wide Population Trends of Marbled Murrelets in Canada Using a Bayesian Hierarchical Model
Source: PLoS One. 2015 Aug 10;10(8):e0134891. doi: 10.1371/journal.pone.0134891 (PMC4530943; doi:10.1371/journal.pone.0134891)
Supplement: S1 Table — Two letter codes refer to conservation regions (Fig 1): CC is Central Mainland Coast, EV is East Vancouver Island, WC is West and North Vancouver Island, HG is Haida Gwaii, NC is North Mainland Coast, and SC is South Mainland Coast. (DOCX) [file pone.0134891.s001.docx]

**Table S1:** Parameter values for a hierarchical Bayesian model estimating temporal trends in Marbled Murrelets from counts using marine radar deployed before dawn at 58 stations in 6 Conservation Regions along the coast of British Columbia. Two letter codes refer to conservation regions (Fig. 1): CC is Central Mainland Coast, EV is east Vancouver Island, WC is West and North Vancouver Island, HG is Haida Gwaii, NC is North Mainland Coast, and SC is South Mainland Coast.

|  |  | Prior |  |  |  | Posterior |  |  |  |  |
| --- | --- | --- | --- | --- | --- | --- | --- | --- | --- | --- |
| Parameter | Effect | Distribution | Mean | Precision |  | Mean | SD | 0.05 | median | 0.95 |
| DOYA[1] | Day of effect (parameter a): CC | Normal | 0 | 10000 |  | 0.001171 | 0.000446 | 0.000565 | 0.001114 | 0.001981 |
| DOYA[2] | Day of effect (parameter a): EV | Normal | 0 | 10000 |  | -0.0002564 | 7.58E-05 | -0.00037 | -0.00026 | -0.00012 |
| DOYA[3] | Day of effect (parameter a): WC | Normal | 0 | 10000 |  | -0.0003496 | 3.97E-05 | -0.00041 | -0.00035 | -0.00028 |
| DOYA[4] | Day of effect (parameter a): HG | Normal | 0 | 10000 |  | -0.0005334 | 6.59E-05 | -0.00063 | -0.00054 | -0.00042 |
| DOYA[5] | Day of effect (parameter a): NC | Normal | 0 | 10000 |  | -0.0002356 | 0.000136 | -0.00041 | -0.00025 | 4.18E-06 |
| DOYA[6] | Day of effect (parameter a): SC | Normal | 0 | 10000 |  | -0.0003262 | 0.000127 | -0.00055 | -0.00031 | -0.00014 |
| DOYC[1] | Day of effect (parameter c): CC | Normal | 0 | 100000000 |  | 18.55 | 5.446 | 10.28 | 18.25 | 27.84 |
| DOYC[2] | Day of effect (parameter c): EV | Normal | 0 | 100000000 |  | 2.018 | 4.957 | -5.518 | 1.774 | 10.38 |
| DOYC[3] | Day of effect (parameter c): WC | Normal | 0 | 100000000 |  | 12.95 | 1.91 | 9.957 | 12.88 | 16.15 |
| DOYC[4] | Day of effect (parameter c): HG | Normal | 0 | 100000000 |  | 6.33 | 1.941 | 3.531 | 6.142 | 9.759 |
| DOYC[5] | Day of effect (parameter c): NC | Normal | 0 | 100000000 |  | -6.81 | 7.343 | -18.1 | -6.892 | 4.879 |
| DOYC[6] | Day of effect (parameter c): SC | Normal | 0 | 100000000 |  | -3.391 | 6.939 | -16.41 | -2.323 | 5.946 |
| SE[1] | Residual error variance: CC | 1/sqrt(gamma) | 0.01 | 0.01 |  | 0.4185 | 0.04402 | 0.353 | 0.4148 | 0.4964 |
| SE[2] | Residual error variance: EC | 1/sqrt(gamma) | 0.01 | 0.01 |  | 0.9272 | 0.04634 | 0.8542 | 0.9254 | 1.007 |
| SE[3] | Residual error variance: WC | 1/sqrt(gamma) | 0.01 | 0.01 |  | 0.6325 | 0.0317 | 0.5828 | 0.6312 | 0.6864 |
| SE[4] | Residual error variance: HG | 1/sqrt(gamma) | 0.01 | 0.01 |  | 0.473 | 0.03503 | 0.4189 | 0.471 | 0.5337 |
| SE[5] | Residual error variance: NC | 1/sqrt(gamma) | 0.01 | 0.01 |  | 0.7623 | 0.05062 | 0.684 | 0.7598 | 0.8497 |
| SE[6] | Residual error variance: SC | 1/sqrt(gamma) | 0.01 | 0.01 |  | 0.5659 | 0.0375 | 0.5081 | 0.5637 | 0.6308 |
| Tilt_effect | Radar tilt | Normal | 0 | 1 |  | 0.09822 | 0.02242 | 0.0652 | 0.09639 | 0.138 |
| Year_SD | Hyper parameter for standard deviation of year effects | Log-normal | 10 | 1.5625 |  | 0.4892 | 0.1005 | 0.3494 | 0.4767 | 0.6725 |
| Year_effect[4] | EV 2003 | Normal | 0 | 0.01 |  | -0.549 | 0.1177 | -0.7133 | -0.5639 | -0.3337 |
| Year_effect[5] | EV 2004 | Normal | 0 | 0.01 |  | 0.4237 | 0.2872 | -0.01341 | 0.4056 | 0.9268 |
| Year_effect[6] | EV 2005 | Normal | 0 | 0.01 |  | -0.1705 | 0.1817 | -0.4406 | -0.1879 | 0.1529 |
| Year_effect[8] | EV 2007 | Normal | 0 | 0.01 |  | -0.6952 | 0.07604 | -0.8025 | -0.7042 | -0.5561 |
| Year_effect[9] | EV 2008 | Normal | 0 | 0.01 |  | -0.7151 | 0.07209 | -0.8167 | -0.7235 | -0.585 |
| Year_effect[10] | EV 2009 | Normal | 0 | 0.01 |  | -0.01211 | 0.2306 | -0.3585 | -0.02807 | 0.3888 |
| Year_effect[11] | EV 2010 | Normal | 0 | 0.01 |  | -0.2833 | 0.1997 | -0.5749 | -0.3026 | 0.07134 |
| Year_effect[12] | EV 2011 | Normal | 0 | 0.01 |  | -0.0519 | 0.2858 | -0.4747 | -0.07775 | 0.454 |
| Year_effect[13] | EV 2012 | Normal | 0 | 0.01 |  | -0.229 | 0.2834 | -0.6298 | -0.2625 | 0.2862 |
| Year_effect[14] | WC 1996 | Normal | 0 | 0.01 |  | 0.2387 | 0.2334 | -0.1017 | 0.2159 | 0.6552 |
| Year_effect[15] | WC 1997 | Normal | 0 | 0.01 |  | 0.4415 | 0.2513 | 0.06732 | 0.422 | 0.8865 |
| Year_effect[16] | WC 1998 | Normal | 0 | 0.01 |  | -0.01093 | 0.1844 | -0.2814 | -0.02666 | 0.3141 |
| Year_effect[17] | WC 1999 | Normal | 0 | 0.01 |  | 0.2618 | 0.2732 | -0.1423 | 0.2363 | 0.7523 |
| Year_effect[18] | WC 2001 | Normal | 0 | 0.01 |  | -0.07018 | 0.1991 | -0.3635 | -0.08958 | 0.288 |
| Year_effect[19] | WC 2002 | Normal | 0 | 0.01 |  | 0.4758 | 0.3132 | 0.0118 | 0.4481 | 1.034 |
| Year_effect[20] | WC 2003 | Normal | 0 | 0.01 |  | 0.2983 | 0.3102 | -0.1604 | 0.2701 | 0.8484 |
| Year_effect[21] | WC 2004 | Normal | 0 | 0.01 |  | 0.5205 | 0.3132 | 0.05617 | 0.4936 | 1.073 |
| Year_effect[22] | WC 2005 | Normal | 0 | 0.01 |  | 0.1553 | 0.302 | -0.2909 | 0.1282 | 0.6894 |
| Year_effect[23] | WC 2006 | Normal | 0 | 0.01 |  | 0.6424 | 0.2225 | 0.305 | 0.6273 | 1.035 |
| Year_effect[25] | WC 2008 | Normal | 0 | 0.01 |  | 0.4124 | 0.3368 | -0.08914 | 0.3821 | 1.008 |
| Year_effect[26] | WC 2009 | Normal | 0 | 0.01 |  | -0.1331 | 0.1297 | -0.3286 | -0.1427 | 0.09568 |
| Year_effect[27] | WC 2010 | Normal | 0 | 0.01 |  | 0.1789 | 0.3005 | -0.2633 | 0.1498 | 0.7134 |
| Year_effect[28] | WC 2011 | Normal | 0 | 0.01 |  | 0.524 | 0.3594 | -0.0104 | 0.4951 | 1.163 |
| Year_effect[29] | WC 2012 | Normal | 0 | 0.01 |  | -0.2103 | 0.2524 | -0.5657 | -0.2436 | 0.2479 |
| Year_effect[30] | WC 2013 | Normal | 0 | 0.01 |  | -0.005827 | 0.3085 | -0.452 | -0.03732 | 0.544 |
| Year_effect[35] | HG 2010 | Normal | 0 | 0.01 |  | 0.1084 | 0.1549 | -0.1251 | 0.09722 | 0.383 |
| Year_effect[38] | NC 2005 | Normal | 0 | 0.01 |  | -0.4959 | 0.07511 | -0.6093 | -0.5019 | -0.3637 |
| Year_effect[41] | SC 2001 | Normal | 0 | 0.01 |  | 0.02416 | 0.1434 | -0.1946 | 0.01423 | 0.2749 |
| Year_effect[42] | SC 2006 | Normal | 0 | 0.01 |  | 0.3823 | 0.1963 | 0.08436 | 0.3703 | 0.7218 |
| Year_effect[44] | SC 2010 | Normal | 0 | 0.01 |  | 0.4062 | 0.2012 | 0.09924 | 0.3921 | 0.7557 |
| muintercept | Hyper parameter for mean of intercepts | Uniform (mean and precision refer to lower and upper bounds) | 1 | 2000 |  | 64.57 | 9.322 | 50.29 | 63.99 | 80.81 |
| sdhintercept | Hyper parameter for standard deviation of intercepts | Log-normal | 3.91 | 1.5625 |  | 0.789 | 0.08823 | 0.6569 | 0.7818 | 0.9445 |
| muhslope | Hyper parameter for mean of slopes | Normal | 0 | 0.0001 |  | -0.1338 | 0.6051 | -1.105 | -0.1461 | 0.8822 |
| sdhslope | Hyper parameter for standard deviation of slopes | Log-normal | 2.30 | 1.5625 |  | 2.851 | 0.7554 | 1.75 | 2.776 | 4.202 |
| intercept[1] | Kwalate | Log-normal | 3.91 | 1.5625 |  | 33.64 | 7.981 | 22.3 | 32.78 | 47.88 |
| intercept[2] | Kakweikan | Log-normal | 3.91 | 1.5625 |  | 83.04 | 19.04 | 55.71 | 81.1 | 116.9 |
| intercept[3] | Wakeman | Log-normal | 3.91 | 1.5625 |  | 56.21 | 13.85 | 36.71 | 54.66 | 80.89 |
| intercept[4] | Wannock | Log-normal | 3.91 | 1.5625 |  | 64.68 | 16.42 | 41.37 | 62.84 | 94.17 |
| intercept[5] | Kilbella | Log-normal | 3.91 | 1.5625 |  | 103.8 | 26.27 | 66.38 | 100.9 | 150.8 |
| intercept[6] | Koeye | Log-normal | 3.91 | 1.5625 |  | 48.75 | 11.92 | 31.63 | 47.44 | 70.21 |
| intercept[7] | Kwatna | Log-normal | 3.91 | 1.5625 |  | 135.7 | 34.74 | 86.06 | 131.9 | 198.2 |
| intercept[8] | Skowquiltz | Log-normal | 3.91 | 1.5625 |  | 46.72 | 12.62 | 29 | 45.16 | 69.56 |
| intercept[9] | Ellerslie | Log-normal | 3.91 | 1.5625 |  | 94.74 | 24.93 | 59.67 | 91.79 | 139.6 |
| intercept[10] | Nekite | Log-normal | 3.91 | 1.5625 |  | 53.88 | 15.4 | 32.64 | 51.9 | 81.6 |
| intercept[11] | Lake Cowichan | Log-normal | 3.91 | 1.5625 |  | 14.03 | 3.428 | 9.2 | 13.62 | 20.28 |
| intercept[12] | Upper Campbell | Log-normal | 3.91 | 1.5625 |  | 43.47 | 9.967 | 29.22 | 42.35 | 61.58 |
| intercept[13] | Comox Lake | Log-normal | 3.91 | 1.5625 |  | 32.21 | 7.502 | 21.54 | 31.35 | 45.84 |
| intercept[14] | Nanaimo Lakes | Log-normal | 3.91 | 1.5625 |  | 5.55 | 1.404 | 3.569 | 5.381 | 8.087 |
| intercept[15] | Sooke Lake | Log-normal | 3.91 | 1.5625 |  | 5.597 | 1.467 | 3.558 | 5.408 | 8.275 |
| intercept[16] | Bedwell | Log-normal | 3.91 | 1.5625 |  | 85.98 | 18.84 | 58.68 | 84.01 | 120 |
| intercept[17] | Bulson | Log-normal | 3.91 | 1.5625 |  | 85.51 | 19.05 | 58 | 83.51 | 119.9 |
| intercept[18] | Kelsey Bay | Log-normal | 3.91 | 1.5625 |  | 25.58 | 5.444 | 17.64 | 25.05 | 35.41 |
| intercept[19] | Klasksish | Log-normal | 3.91 | 1.5625 |  | 103.1 | 27.23 | 64.92 | 99.57 | 152.7 |
| intercept[20] | Megin | Log-normal | 3.91 | 1.5625 |  | 83.77 | 19.62 | 55.64 | 81.62 | 119.3 |
| intercept[21] | Moyeha | Log-normal | 3.91 | 1.5625 |  | 126.7 | 28.52 | 85.48 | 123.7 | 178.1 |
| intercept[22] | Nitinat | Log-normal | 3.91 | 1.5625 |  | 63.12 | 16.25 | 40.26 | 61.1 | 92.75 |
| intercept[23] | Power | Log-normal | 3.91 | 1.5625 |  | 99.41 | 24.87 | 64.2 | 96.52 | 144.6 |
| intercept[24] | Tahsis | Log-normal | 3.91 | 1.5625 |  | 53.48 | 13.04 | 34.9 | 52.05 | 77.09 |
| intercept[25] | Tahsish | Log-normal | 3.91 | 1.5625 |  | 150.4 | 37.45 | 97.39 | 146 | 218.7 |
| intercept[26] | Toquart | Log-normal | 3.91 | 1.5625 |  | 104.1 | 27.69 | 65.28 | 100.7 | 154.5 |
| intercept[27] | Watta | Log-normal | 3.91 | 1.5625 |  | 61.15 | 13.48 | 41.6 | 59.75 | 85.46 |
| intercept[28] | Bigsby | Log-normal | 3.91 | 1.5625 |  | 57.76 | 11.24 | 41.14 | 56.81 | 77.74 |
| intercept[29] | Botany | Log-normal | 3.91 | 1.5625 |  | 42.82 | 8.261 | 30.62 | 42.08 | 57.5 |
| intercept[30] | Dawson Inlet | Log-normal | 3.91 | 1.5625 |  | 44.41 | 8.007 | 32.43 | 43.79 | 58.54 |
| intercept[31] | Fairfax | Log-normal | 3.91 | 1.5625 |  | 39.27 | 7.855 | 27.74 | 38.55 | 53.25 |
| intercept[32] | Huston | Log-normal | 3.91 | 1.5625 |  | 38.2 | 7.141 | 27.53 | 37.62 | 50.9 |
| intercept[33] | Hutton | Log-normal | 3.91 | 1.5625 |  | 30.11 | 5.789 | 21.51 | 29.6 | 40.37 |
| intercept[34] | Klunkwoi | Log-normal | 3.91 | 1.5625 |  | 61.52 | 11.49 | 44.32 | 60.61 | 81.81 |
| intercept[35] | Lagoon | Log-normal | 3.91 | 1.5625 |  | 46.49 | 8.853 | 33.35 | 45.73 | 62.21 |
| intercept[36] | Long | Log-normal | 3.91 | 1.5625 |  | 75.45 | 13.56 | 55.11 | 74.33 | 99.4 |
| intercept[37] | Port Channel | Log-normal | 3.91 | 1.5625 |  | 213.7 | 38.87 | 155.7 | 210.5 | 282.5 |
| intercept[38] | Tartu | Log-normal | 3.91 | 1.5625 |  | 59.02 | 11.8 | 41.74 | 57.89 | 80.05 |
| intercept[39] | Windy Bay | Log-normal | 3.91 | 1.5625 |  | 98.7 | 22.55 | 66 | 96.44 | 139.1 |
| intercept[40] | Aaltanhash | Log-normal | 3.91 | 1.5625 |  | 35.12 | 10.34 | 20.71 | 33.82 | 54.01 |
| intercept[41] | Brim River | Log-normal | 3.91 | 1.5625 |  | 29.97 | 5.96 | 21.19 | 29.44 | 40.57 |
| intercept[42] | East Inlet | Log-normal | 3.91 | 1.5625 |  | 63.93 | 15.68 | 41.72 | 62.11 | 92.43 |
| intercept[43] | Gilttoyees | Log-normal | 3.91 | 1.5625 |  | 51.67 | 12.16 | 34.28 | 50.3 | 73.67 |
| intercept[44] | Green | Log-normal | 3.91 | 1.5625 |  | 73.91 | 18.51 | 47.43 | 71.9 | 107.3 |
| intercept[45] | Khutze | Log-normal | 3.91 | 1.5625 |  | 57.56 | 13.45 | 38.31 | 56.04 | 81.85 |
| intercept[46] | Khutzeymateen River | Log-normal | 3.91 | 1.5625 |  | 66.63 | 16.92 | 42.76 | 64.64 | 97.35 |
| intercept[47] | Kwinamass River | Log-normal | 3.91 | 1.5625 |  | 151.4 | 36.23 | 100 | 147.3 | 217 |
| intercept[48] | Toon River | Log-normal | 3.91 | 1.5625 |  | 122.1 | 29.85 | 79.49 | 118.8 | 176.2 |
| intercept[49] | Brem | Log-normal | 3.91 | 1.5625 |  | 88.12 | 20.31 | 59.03 | 85.91 | 124.6 |
| intercept[50] | Brittain | Log-normal | 3.91 | 1.5625 |  | 15.56 | 3.752 | 10.21 | 15.12 | 22.34 |
| intercept[51] | Deserted | Log-normal | 3.91 | 1.5625 |  | 23.07 | 5.82 | 14.8 | 22.39 | 33.67 |
| intercept[52] | Forbes | Log-normal | 3.91 | 1.5625 |  | 43.71 | 10.17 | 29.2 | 42.55 | 62.17 |
| intercept[53] | Orford | Log-normal | 3.91 | 1.5625 |  | 56.58 | 12.55 | 38.42 | 55.26 | 79.25 |
| intercept[54] | Quatam | Log-normal | 3.91 | 1.5625 |  | 42.25 | 9.265 | 28.93 | 41.26 | 59.02 |
| intercept[55] | Skakwa | Log-normal | 3.91 | 1.5625 |  | 47.83 | 11.03 | 32.01 | 46.65 | 67.77 |
| intercept[56] | Southgate | Log-normal | 3.91 | 1.5625 |  | 16.25 | 4.045 | 10.54 | 15.76 | 23.61 |
| intercept[57] | Toba | Log-normal | 3.91 | 1.5625 |  | 58.17 | 12.86 | 39.53 | 56.79 | 81.29 |
| intercept[58] | Vancouver | Log-normal | 3.91 | 1.5625 |  | 29.31 | 6.704 | 19.7 | 28.59 | 41.41 |
| slope[1] | Kwalate | Normal | 0 | 2.30 |  | -0.7229 | 1.369 | -3.063 | -0.6342 | 1.354 |
| slope[2] | Kakweikan | Normal | 0 | 2.30 |  | 2.49 | 2.171 | -0.9931 | 2.456 | 6.108 |
| slope[3] | Wakeman | Normal | 0 | 2.30 |  | -0.1525 | 1.856 | -3.291 | -0.084 | 2.773 |
| slope[4] | Wannock | Normal | 0 | 2.30 |  | 0.5007 | 1.911 | -2.582 | 0.4986 | 3.623 |
| slope[5] | Kilbella | Normal | 0 | 2.30 |  | -0.6521 | 2.318 | -4.486 | -0.631 | 3.09 |
| slope[6] | Koeye | Normal | 0 | 2.30 |  | 1.814 | 1.515 | -0.656 | 1.801 | 4.28 |
| slope[7] | Kwatna | Normal | 0 | 2.30 |  | 2.085 | 2.635 | -2.052 | 2 | 6.535 |
| slope[8] | Skowquiltz | Normal | 0 | 2.30 |  | 2.834 | 1.715 | 0.03398 | 2.82 | 5.676 |
| slope[9] | Ellerslie | Normal | 0 | 2.30 |  | 3.89 | 2.541 | -0.06067 | 3.784 | 8.245 |
| slope[10] | Nekite | Normal | 0 | 2.30 |  | -0.7123 | 2.811 | -5.357 | -0.6925 | 3.866 |
| slope[11] | Lake Cowichan | Normal | 0 | 2.30 |  | -1.344 | 0.6468 | -2.437 | -1.326 | -0.3216 |
| slope[12] | Upper Campbell | Normal | 0 | 2.30 |  | 1.414 | 1.891 | -1.464 | 1.287 | 4.723 |
| slope[13] | Comox Lake | Normal | 0 | 2.30 |  | -2.35 | 1.355 | -4.607 | -2.325 | -0.1768 |
| slope[14] | Nanaimo Lakes | Normal | 0 | 2.30 |  | -1.131 | 0.4157 | -1.873 | -1.085 | -0.542 |
| slope[15] | Sooke Lake | Normal | 0 | 2.30 |  | -0.8247 | 0.3864 | -1.498 | -0.8004 | -0.2428 |
| slope[16] | Bedwell | Normal | 0 | 2.30 |  | -1.336 | 1.799 | -4.358 | -1.285 | 1.535 |
| slope[17] | Bulson | Normal | 0 | 2.30 |  | -1.446 | 1.833 | -4.541 | -1.404 | 1.467 |
| slope[18] | Kelsey Bay | Normal | 0 | 2.30 |  | 0.8527 | 1.091 | -0.9048 | 0.8428 | 2.667 |
| slope[19] | Klasksish | Normal | 0 | 2.30 |  | -1.578 | 2.657 | -6.1 | -1.485 | 2.544 |
| slope[20] | Megin | Normal | 0 | 2.30 |  | -1.666 | 1.962 | -5.011 | -1.577 | 1.368 |
| slope[21] | Moyeha | Normal | 0 | 2.30 |  | 1.598 | 2.412 | -2.156 | 1.513 | 5.717 |
| slope[22] | Nitinat | Normal | 0 | 2.30 |  | -1.975 | 2.599 | -6.423 | -1.885 | 2.066 |
| slope[23] | Power | Normal | 0 | 2.30 |  | -0.1686 | 2.481 | -4.248 | -0.1553 | 3.889 |
| slope[24] | Tahsis | Normal | 0 | 2.30 |  | -0.1533 | 2.019 | -3.478 | -0.1462 | 3.15 |
| slope[25] | Tahsish | Normal | 0 | 2.30 |  | -0.03355 | 2.832 | -4.632 | -0.06053 | 4.659 |
| slope[26] | Toquart | Normal | 0 | 2.30 |  | 0.4914 | 2.82 | -4.02 | 0.4392 | 5.212 |
| slope[27] | Watta | Normal | 0 | 2.30 |  | 2.681 | 1.516 | 0.3301 | 2.6 | 5.303 |
| slope[28] | Bigsby | Normal | 0 | 2.30 |  | -1.969 | 2.319 | -5.916 | -1.849 | 1.645 |
| slope[29] | Botany | Normal | 0 | 2.30 |  | -2.83 | 2.212 | -6.547 | -2.794 | 0.7313 |
| slope[30] | Dawson Inlet | Normal | 0 | 2.30 |  | 1.111 | 2.135 | -2.239 | 1.012 | 4.753 |
| slope[31] | Fairfax | Normal | 0 | 2.30 |  | -5.453 | 2.718 | -10.15 | -5.296 | -1.27 |
| slope[32] | Huston | Normal | 0 | 2.30 |  | -4.45 | 2.051 | -7.969 | -4.34 | -1.26 |
| slope[33] | Hutton | Normal | 0 | 2.30 |  | -2.531 | 1.589 | -5.242 | -2.469 | -0.03998 |
| slope[34] | Klunkwoi | Normal | 0 | 2.30 |  | 5.003 | 2.526 | 1.122 | 4.86 | 9.39 |
| slope[35] | Lagoon | Normal | 0 | 2.30 |  | -0.01088 | 2.031 | -3.37 | 0.002149 | 3.322 |
| slope[36] | Long | Normal | 0 | 2.30 |  | -2.505 | 2.446 | -6.682 | -2.392 | 1.275 |
| slope[37] | Port Channel | Normal | 0 | 2.30 |  | 0.8307 | 2.982 | -3.837 | 0.7301 | 5.918 |
| slope[38] | Tartu | Normal | 0 | 2.30 |  | -1.215 | 2.315 | -5.054 | -1.164 | 2.508 |
| slope[39] | Windy Bay | Normal | 0 | 2.30 |  | -0.7942 | 2.917 | -5.641 | -0.7489 | 3.875 |
| slope[40] | Aaltanhash | Normal | 0 | 2.30 |  | 0.6728 | 1.749 | -2.284 | 0.7186 | 3.447 |
| slope[41] | Brim River | Normal | 0 | 2.30 |  | 0.2898 | 1.288 | -1.813 | 0.2856 | 2.415 |
| slope[42] | East Inlet | Normal | 0 | 2.30 |  | 0.03524 | 2.614 | -4.16 | -0.00015 | 4.385 |
| slope[43] | Gilttoyees | Normal | 0 | 2.30 |  | 0.3291 | 2.122 | -3.122 | 0.3314 | 3.805 |
| slope[44] | Green | Normal | 0 | 2.30 |  | -0.7991 | 2.516 | -4.962 | -0.7825 | 3.305 |
| slope[45] | Khutze | Normal | 0 | 2.30 |  | 2.53 | 1.86 | -0.4478 | 2.486 | 5.648 |
| slope[46] | Khutzeymateen River | Normal | 0 | 2.30 |  | 2.441 | 2.587 | -1.537 | 2.314 | 6.881 |
| slope[47] | Kwinamass River | Normal | 0 | 2.30 |  | 3.23 | 3.497 | -1.786 | 2.859 | 9.618 |
| slope[48] | Toon River | Normal | 0 | 2.30 |  | -0.08865 | 2.874 | -4.735 | -0.1305 | 4.689 |
| slope[49] | Brem | Normal | 0 | 2.30 |  | -0.2759 | 2.201 | -3.877 | -0.2648 | 3.322 |
| slope[50] | Brittain | Normal | 0 | 2.30 |  | -1.582 | 0.6145 | -2.686 | -1.52 | -0.69 |
| slope[51] | Deserted | Normal | 0 | 2.30 |  | -2.1 | 1.214 | -4.284 | -1.984 | -0.3271 |
| slope[52] | Forbes | Normal | 0 | 2.30 |  | -1.948 | 1.445 | -4.4 | -1.89 | 0.3186 |
| slope[53] | Orford | Normal | 0 | 2.30 |  | 1.24 | 1.757 | -1.545 | 1.202 | 4.192 |
| slope[54] | Quatam | Normal | 0 | 2.30 |  | -1.497 | 1.416 | -3.863 | -1.468 | 0.7376 |
| slope[55] | Skakwa | Normal | 0 | 2.30 |  | 2.039 | 1.611 | -0.5015 | 1.991 | 4.734 |
| slope[56] | Southgate | Normal | 0 | 2.30 |  | -1.451 | 0.8112 | -2.888 | -1.372 | -0.286 |
| slope[57] | Toba | Normal | 0 | 2.30 |  | -0.08168 | 1.654 | -2.775 | -0.08547 | 2.637 |
| slope[58] | Vancouver | Normal | 0 | 2.30 |  | -0.4544 | 1.103 | -2.299 | -0.4366 | 1.31 |
