# Supplementary material for: Estimation of Coast-Wide Population Trends of Marbled Murrelets in Canada Using a Bayesian Hierarchical Model
Source: PLoS One. 2015 Aug 10;10(8):e0134891. doi: 10.1371/journal.pone.0134891 (PMC4530943; doi:10.1371/journal.pone.0134891)
Supplement: S2 Table — (DOCX) [file pone.0134891.s002.docx]

S9 Table. Names and locations for radar monitoring stations for Marbled Murrelets at 6 Conservation Regions on the coast of British Columbia, Canada.

| Station | Region | Latitude | Longitude | Mean Number of Incoming Murrelets |
| --- | --- | --- | --- | --- |
| Ellerslie | Central Mainland Coast | 52.514797 | -127.992465 | 516.1 |
| Kakweikan | Central Mainland Coast | 50.798246 | -126.014045 | 443.6 |
| Kilbella | Central Mainland Coast | 51.694590 | -127.357965 | 526.6 |
| Koeye | Central Mainland Coast | 51.772766 | -127.877367 | 210.9 |
| Kwalate | Central Mainland Coast | 50.779357 | -125.683316 | 144.6 |
| Kwatna | Central Mainland Coast | 52.103921 | -127.395257 | 698.0 |
| Nekite 2 | Central Mainland Coast | 51.380570 | -127.116947 | 315.0 |
| Skowquiltz | Central Mainland Coast | 52.593799 | -127.166172 | 223.4 |
| Wakeman | Central Mainland Coast | 50.989633 | -126.427556 | 308.7 |
| Wannock | Central Mainland Coast | 51.670931 | -127.269719 | 342.3 |
| Comox Lake | East Vancouver Island | 49.580653 | -125.186696 | 63.4 |
| Kelsey Bay | East Vancouver Island | 50.397579 | -125.960798 | 60.7 |
| Lake Cowichan | East Vancouver Island | 48.909332 | -124.338493 | 20.2 |
| Nanaimo Lakes | East Vancouver Island | 49.101396 | -124.396169 | 10.4 |
| Sooke Lake | East Vancouver Island | 48.558567 | -123.702649 | 11.6 |
| Upper Campbell | East Vancouver Island | 49.881821 | -125.680987 | 95.8 |
| Bigsby | Haida Gwaii | 52.618214 | -131.716228 | 133.9 |
| Botany | Haida Gwaii | 52.737776 | -131.940637 | 73.6 |
| Dawson Inlet | Haida Gwaii | 53.170995 | -132.487253 | 97.3 |
| Fairfax | Haida Gwaii | 52.740007 | -131.986846 | 79.8 |
| Huston | Haida Gwaii | 52.268601 | -131.268213 | 83.3 |
| Hutton | Haida Gwaii | 52.500972 | -131.562659 | 64.4 |
| Klunkwoi | Haida Gwaii | 52.735447 | -131.817262 | 177.0 |
| Lagoon | Haida Gwaii | 52.923746 | -131.924956 | 105.3 |
| Long | Haida Gwaii | 53.205070 | -132.276281 | 121.9 |
| Port Chanal | Haida Gwaii | 53.593937 | -132.891784 | 420.5 |
| Tartu | Haida Gwaii | 53.470113 | -132.674963 | 116.0 |
| Windy Bay | Haida Gwaii | 52.690164 | -131.447212 | 164.4 |
| Aaltanhash | North Mainland Coast | 53.141337 | -128.478335 | 40.3 |
| Baker Inlet | North Mainland Coast | 53.813380 | -129.855547 | 32.8 |
| Brim River | North Mainland Coast | 53.508278 | -128.369907 | 88.3 |
| East Inlet | North Mainland Coast | 53.704322 | -129.714727 | 68.1 |
| Gilttoyees | North Mainland Coast | 53.890893 | -128.990612 | 114.5 |
| Green | North Mainland Coast | 52.939740 | -128.410643 | 88.6 |
| Khutze | North Mainland Coast | 53.085227 | -128.434412 | 136.4 |
| Khutzeymateen River | North Mainland Coast | 54.602367 | -129.936134 | 172.6 |
| Kwinamass River | North Mainland Coast | 54.783531 | -130.170015 | 387.7 |
| Toon River | North Mainland Coast | 54.503042 | -130.001929 | 177.5 |
| Brem | South Mainland Coast | 50.433368 | -124.665763 | 308.0 |
| Brittain | South Mainland Coast | 49.993273 | -124.007556 | 60.8 |
| Deserted | South Mainland Coast | 50.090364 | -123.745677 | 65.2 |
| Forbes | South Mainland Coast | 50.246366 | -124.591869 | 135.5 |
| Orford | South Mainland Coast | 50.595443 | -124.864637 | 216.9 |
| Quatam | South Mainland Coast | 50.379349 | -124.942451 | 149.5 |
| Skwakwa | South Mainland Coast | 50.208557 | -123.891518 | 180.5 |
| Southgate | South Mainland Coast | 50.894692 | -124.793872 | 51.1 |
| Toba | South Mainland Coast | 50.482536 | -124.386341 | 214.9 |
| Vancouver | South Mainland Coast | 49.922440 | -123.875440 | 111.7 |
| Bedwell | West and North Vancouver Island | 49.355840 | -125.774916 | 304.4 |
| Bulson | West and North Vancouver Island | 49.254809 | -125.732293 | 287.4 |
| Klaskish | West and North Vancouver Island | 50.241691 | -127.761849 | 380.4 |
| Megin | West and North Vancouver Island | 49.435120 | -126.079282 | 331.0 |
| Moyeha | West and North Vancouver Island | 49.409843 | -125.913398 | 519.7 |
| Nitinat | West and North Vancouver Island | 48.807412 | -124.676069 | 248.8 |
| Power | West and North Vancouver Island | 50.174758 | -127.474705 | 301.8 |
| Tahsis | West and North Vancouver Island | 49.974467 | -126.664187 | 169.0 |
| Tahsish Inlet | West and North Vancouver Island | 50.134933 | -127.102418 | 371.7 |
| Toquart | West and North Vancouver Island | 49.022657 | -125.357814 | 363.5 |
| Watta | West and North Vancouver Island | 49.428289 | -126.039278 | 363.8 |
