# Supplementary material for: Estimation of Coast-Wide Population Trends of Marbled Murrelets in Canada Using a Bayesian Hierarchical Model
Source: PLoS One. 2015 Aug 10;10(8):e0134891. doi: 10.1371/journal.pone.0134891 (PMC4530943; doi:10.1371/journal.pone.0134891)
Supplement: S1 Text — (DOCX) [file pone.0134891.s004.docx]

**# HIERARCHICAL REGRESSION MODEL PREDICTING PRE-DAWN RADAR COUNTS**

# Murdoch McAllister (m.mcallister@fisheries.ubc.ca)

# slope and intercept are treated as hierarchical

# Year covariate is mean centered (X-Xbar)

# Reduced set of year effects estimated

# precision is estimated by region

#DOY effects different across regions and setting the constant at zero

#variance in year effect estimated - using DOY unadjusted - 182

#estimating DOYC by region as adjustment from 182 centre point

#prior mean for SD in year effect set at 10

#tilt treated as a covariate

#evaluate the probability of each observed radar count given the model

for (i in 1:943){

Obs_Predawn[i]~dlnorm(lpredD[i], precD[Region[i]])

lpredD[i]<-log(predD[i])

predD[i]<- max(predDx[i],1)

#predict the pred-dawn count given the year, etc.

predDx[i]<-(intercept[Site[i]]+slope[Site[i]]*(Year[i]-Mean_Year[i]))*(1+Year_effect[Year_ind[i]])*DOYE[i]*(1+Tilt_effect*Tilt[i])

#predict the date of year effect by region

DOYE[i]<- 1+ DOYA[Region[i]]*(DOY[i]+DOYC[Region[i]])*(DOY[i]+DOYC[Region[i]]) #+DOYB[Region[i]]*(DOY[i]+DOYC[Region[i]])

}

#Predict the abundance for the 1st yr for site #1

DOYS <- 10 #determine a standard DOY

predD_P[1]<- max(predD_Px[1],1)

predD_Px[1]<-(intercept[Sited[1]]+slope[Sited[1]]*(Yeard[1]-Mean_yeard[1]))*(1+Year_effect[Yearc[1]])*(1+Tilt_effect*25) * DOYEP[1]

#compute DOY effect

DOYEP[1]<- 1+ DOYA[Regiond[1]]*(DOYS+DOYC[Regiond[1]])*(DOYS+DOYC[Regiond[1]])

delta_s[1] <- 0

#Predict the abundance for each observed yr for each site

for (i in 2:269){

predD_P[i]<- max(predD_Px[i],1)

predD_Px[i]<-(intercept[Sited[i]]+slope[Sited[i]]*(Yeard[i]-Mean_yeard[i]))*(1+Year_effect[Yearc[i]])*(1+Tilt_effect*25) * DOYEP[i]

#compute DOY effect

DOYEP[i]<- 1+ DOYA[Regiond[i]]*(DOYS+DOYC[Regiond[i]])*(DOYS+DOYC[Regiond[i]])

#compute the relative difference in abund. bet. ea. successive observed time

delta_s[i] <- (predD_P[i] - predD_P[i-1])/ (predD_P[i-1]*(Yeard[i]-Yeard[i-1]))

}

#compute province-wide mean annual change in abundance

delta_s_all <- mean(delta_s_av[1:58])

#compute probability that there is a province wide decline

P_value_all <- 1- step(delta_s_all)

#lafit<-log(0.000004) #

taua<-1/(0.01*0.01)

taub<-1/(0.0001*0.0001)

tauc<-1/(10*10)

for (i in 1:6){

DOYA[i]~dnorm(0,taua)

DOYA_prior[i]~dnorm(0,taua)

# DOYB[i]~dnorm(0,taub)

# DOYB_prior[i]~dnorm(0,taub)

# DOYC[i]<- 0 #-DOYCp[i] # the constant in the 2nd O polynomial is set to zero

DOYC[i]~dnorm(0,tauc)

DOYC_prior[i]~dnorm(0,tauc)

precD[i]~dgamma(0.01,0.01) #prior on the precision in the data

SE[i] <- 1/ sqrt(precD[i])

#compute probability of decline in each region

#method #1

pval_delta_Ra[i] <- 1-step(delta_Rava[i])

#method #2

delta_Ravb[i] <- mean(delta_s_av[minReg[i]:maxReg[i]])

pval_delta_Rb[i] <- 1-step(delta_Ravb[i])

}

#prior put on sd in year effects

Year_medSD < - 10

lYear_medSD<- log(Year_medSD)

tau_Year<- 1/ (0.8*0.8)

Year_SD~dlnorm(lYear_medSD, tau_Year)

Year_SDp~dlnorm(lYear_medSD, tau_Year)

Year_tau<- 1/ (Year_SD*Year_SD)

#Determine year effect priors

#Central Coast 1-3

#predict region wide count for CC in each year

predD_R[1]<- max(predD_Rx[1],1)

predD_Rx[1]<-(muinterceptR[1]+muslopeR[1]*(YearR[1]-MeanYR[1]))*(1+Year_effect[1])*(1+Tilt_effect*25)*DOYER[1]

DOYER[1]<- 1+ DOYA[1]*(DOYS+DOYC[1])*(DOYS+DOYC[1])

delta_R[1] <- 0

for (i in 2: 3) {

predD_R[i]<- max(predD_Rx[i],1)

predD_Rx[i]<-(muinterceptR[1]+muslopeR[1]*(YearR[i]-MeanYR[1]))*(1+Year_effect[i])*(1+Tilt_effect*25)*DOYER[1]

delta_R[i] <- (predD_R[i] - predD_R[i-1]) / (predD_R[i-1] * (YearR[i]-YearR[i-1]))

}

delta_Rava[1] <- mean(delta_R[2:3]) #calc the average rate of change by regio

Year_effect[1]<-0

#Year_effect[2]~dnorm(0,Year_tau)

Year_effect[2]<-0

Year_effect[3]<-0

#East Vancouver Island 4-13

predD_R[4]<- max(predD_Rx[4],1)

predD_Rx[4]<-(muinterceptR[2]+muslopeR[2]*(YearR[4]-MeanYR[2]))*(1+Year_effect[4])*(1+Tilt_effect*25)*DOYER[2]

DOYER[2]<- 1+ DOYA[2]*(DOYS+DOYC[2])*(DOYS+DOYC[2])

for (i in 5: 13) {

predD_R[i]<- max(predD_Rx[i],1)

predD_Rx[i]<-(muinterceptR[2]+muslopeR[2]*(YearR[i]-MeanYR[2]))*(1+Year_effect[i])*(1+Tilt_effect*25)*DOYER[2]

delta_R[i] <- (predD_R[i] - predD_R[i-1]) /(predD_R[i-1] * (YearR[i]-YearR[i-1]))

}

delta_Rava[2] <- mean(delta_R[5:13])

for (i in 4:6){

Year_effect[i]~dnorm(0,Year_tau)

}

Year_effect[7]<-0 #OK

#West coast 14-30

predD_R[14]<- max(predD_Rx[14],1)

predD_Rx[14]<-(muinterceptR[3]+muslopeR[3]*(YearR[14]-MeanYR[3]))*(1+Year_effect[14])*(1+Tilt_effect*25)*DOYER[3]

DOYER[3]<- 1+ DOYA[3]*(DOYS+DOYC[3])*(DOYS+DOYC[3])

for (i in 15: 30) {

predD_R[i]<- max(predD_Rx[i],1)

predD_Rx[i]<-(muinterceptR[3]+muslopeR[3]*(YearR[i]-MeanYR[3]))*(1+Year_effect[i])*(1+Tilt_effect*25)*DOYER[3]

delta_R[i] <- (predD_R[i] - predD_R[i-1]) / (predD_R[i-1] * (YearR[i]-YearR[i-1]))

}

delta_Rava[3] <- mean(delta_R[15:30])

for (i in 8:23){

Year_effect[i]~dnorm(0,Year_tau)

}

Year_effect[24]<-0 #was before 14

for (i in 25:30){

Year_effect[i]~dnorm(0,Year_tau)

}

#Haida Gwai 31-35

predD_R[31]<- max(predD_Rx[31],1)

predD_Rx[31]<-(muinterceptR[4]+muslopeR[4]*(YearR[31]-MeanYR[4]))*(1+Year_effect[31])*(1+Tilt_effect*25)*DOYER[4]

DOYER[4]<- 1+ DOYA[4]*(DOYS+DOYC[4])*(DOYS+DOYC[4])

for (i in 32: 35) {

predD_R[i]<- max(predD_Rx[i],1)

predD_Rx[i]<-(muinterceptR[4]+muslopeR[4]*(YearR[i]-MeanYR[4]))*(1+Year_effect[i])*(1+Tilt_effect*25)*DOYER[4]

delta_R[i] <- (predD_R[i] - predD_R[i-1]) /(predD_R[i-1] * (YearR[i]-YearR[i-1]))

}

delta_Rava[4] <- mean(delta_R[32:35])

Year_effect[31]<-0

for (i in 32:34){

Year_effect[i] <-0

}

Year_effect[35]~dnorm(0,Year_tau)

#North coast 36-39

predD_R[36]<- max(predD_Rx[36],1)

predD_Rx[36]<-(muinterceptR[5]+muslopeR[5]*(YearR[36]-MeanYR[5]))*(1+Year_effect[36])*(1+Tilt_effect*25)*DOYER[5]

DOYER[5]<- 1+ DOYA[5]*(DOYS+DOYC[5])*(DOYS+DOYC[5])

for (i in 37: 39) {

predD_R[i]<- max(predD_Rx[i],1)

predD_Rx[i]<-(muinterceptR[5]+muslopeR[5]*(YearR[i]-MeanYR[5]))*(1+Year_effect[i])*(1+Tilt_effect*25)*DOYER[5]

delta_R[i] <- (predD_R[i] - predD_R[i-1]) /(predD_R[i-1] * (YearR[i]-YearR[i-1]))

}

delta_Rava[5] <- mean(delta_R[37:39])

Year_effect[36]<-0

Year_effect[37]<-0

Year_effect[38]~dnorm(0,Year_tau)

Year_effect[39]<-0

#South coast 40-44

predD_R[40]<- max(predD_Rx[40],1)

predD_Rx[40]<-(muinterceptR[6]+muslopeR[6]*(YearR[40]-MeanYR[6]))*(1+Year_effect[40])*(1+Tilt_effect*25)*DOYER[6]

DOYER[6]<- 1+ DOYA[6]*(DOYS+DOYC[6])*(DOYS+DOYC[6])

for (i in 41: 44) {

predD_R[i]<- max(predD_Rx[i],1)

predD_Rx[i]<-(muinterceptR[6]+muslopeR[6]*(YearR[i]-MeanYR[6]))*(1+Year_effect[i])*(1+Tilt_effect*25)*DOYER[6]

delta_R[i] <- (predD_R[i] - predD_R[i-1]) / (predD_R[i-1] * (YearR[i]-YearR[i-1]))

}

delta_Rava[6] <- mean(delta_R[41:44])

Year_effect[40]<-0

for (i in 41:42){

Year_effect[i]~dnorm(0,Year_tau)

}

Year_effect[43]<-0

Year_effect[44]~dnorm(0,Year_tau)

muhslope~dnorm(0,0.0001) #hyper prior for mean of slopes

muhslopep~dnorm(0,0.0001) #hyper prior for mean of slopes

tauhslope<- 1/(sdhslope*sdhslope) #computation of prior prec. in slope

sdhslope~dlnorm(lsdh_mu,tau_sdh) #hyper prior for sd of slopes

sdhslopep~dlnorm(lsdh_mu,tau_sdh) #hyper prior for sd of slopes

sdh_mu<-10

lsdh_mu<-log(sdh_mu)

sdh_sd<-0.8

tau_sdh<- 1/(sdh_sd*sdh_sd)

#slope to be treated as a hierarchical parameter across sites

for (i in 1:58){

slope[i]~dnorm(muhslope,tauhslope)

intercept[i]~dlnorm(lmedinterc,tauint)

rate[i]<-slope[i]/intercept[i]

Pvalue_rate[i] <- 1-step(rate[i]) #calc prob of a decrease

#calc. the average rate of change per site.

delta_s_av[i] <- mean(delta_s[minSite[i]: maxSite[i]])

#calc. prob. of decline in each site

P_value_delta_s[i] <- 1- step(delta_s_av[i])

}

#lmedinterc<-log(50)

#tauint<-1/(0.8*0.8)

lmedinterc<-log(medinterc)

#medinterc~dlnorm(lmedintercp,tauintp) #hyperprior for prior med for intercept

#medintercp~dlnorm(lmedintercp,tauintp) #hyperprior for prior med for intercept

medinterc~dunif(1, 2000)

medintercp~dunif(1, 2000)

lmedintercp<-log(50)

tauintp<-1/(0.8*0.8)

tauint<-1/(sdhintercept*sdhintercept) #hyperprior for prior prec of intercept

sdhintercept~dlnorm(lsdinth_mu,tau_sdinth) #hyper prior for sd of intercept

sdhinterceptp~dlnorm(lsdinth_mu,tau_sdinth) #hyper prior for sd of intercepts

sdinth_mu<-50 #should be centred at a much lower value, e.g., at 1

lsdinth_mu<-log(sdinth_mu)

sdinth_sd<-0.8

tau_sdinth<- 1/(sdinth_sd*sdinth_sd)

#prior density for tilt effect

Tilt_effect~dnorm(0,1)

Tilt_effect_prior~dnorm(0,1)

#calc. the averages for slopes and intercepts across sites and regions

muslopeR[1]<- mean(slope[1:10])

muslopeR[2]<-mean(slope[11:16])

muslopeR[3]<-mean(slope[17:27])

muslopeR[4]<- mean(slope[28:39])

muslopeR[5]<-mean(slope[40:48])

muslopeR[6]<-mean(slope[49:58])

muinterceptR[1]<- mean(intercept[1:10])

muinterceptR[2]<-mean(intercept[11:16])

muinterceptR[3]<-mean(intercept[17:27])

muinterceptR[4]<- mean(intercept[28:39])

muinterceptR[5]<-mean(intercept[40:48])

muinterceptR[6]<-mean(intercept[49:58])

murateR[1]<- mean(rate[1:10])

murateR[2]<-mean(rate[11:16])

murateR[3]<-mean(rate[17:27])

murateR[4]<- mean(rate[28:39])

murateR[5]<-mean(rate[40:48])

murateR[6]<-mean(rate[49:58])

for (i in 1:6) {

Pvalue_rateR[i] <- 1-step(murateR[i]) #calc prob of a decrease

}

muslopep<- abs(muslope)

muratep<- abs(murate)

muslope<-mean(slope[]) #take mean of slopes across sites

sdslope<-sd(slope[]) #take sd of slope across sites

murate<-mean(rate[]) #take mean of rates across sites

Pvalue_murate <- 1-step(murate)

sdrate<-sd(rate[]) #take sd of rate across sites

CVslope<-sdslope/muslopep

CVrate<-sdrate/muratep

muintercept<-mean(intercept[])

sdintercept<-sd(intercept[])

CVintercept<-sdintercept/muintercept

}
