# Supplementary figures and images for: Estimation of Coast-Wide Population Trends of Marbled Murrelets in Canada Using a Bayesian Hierarchical Model
Source: PLoS One. 2015 Aug 10;10(8):e0134891. doi: 10.1371/journal.pone.0134891 (PMC4530943; doi:10.1371/journal.pone.0134891)

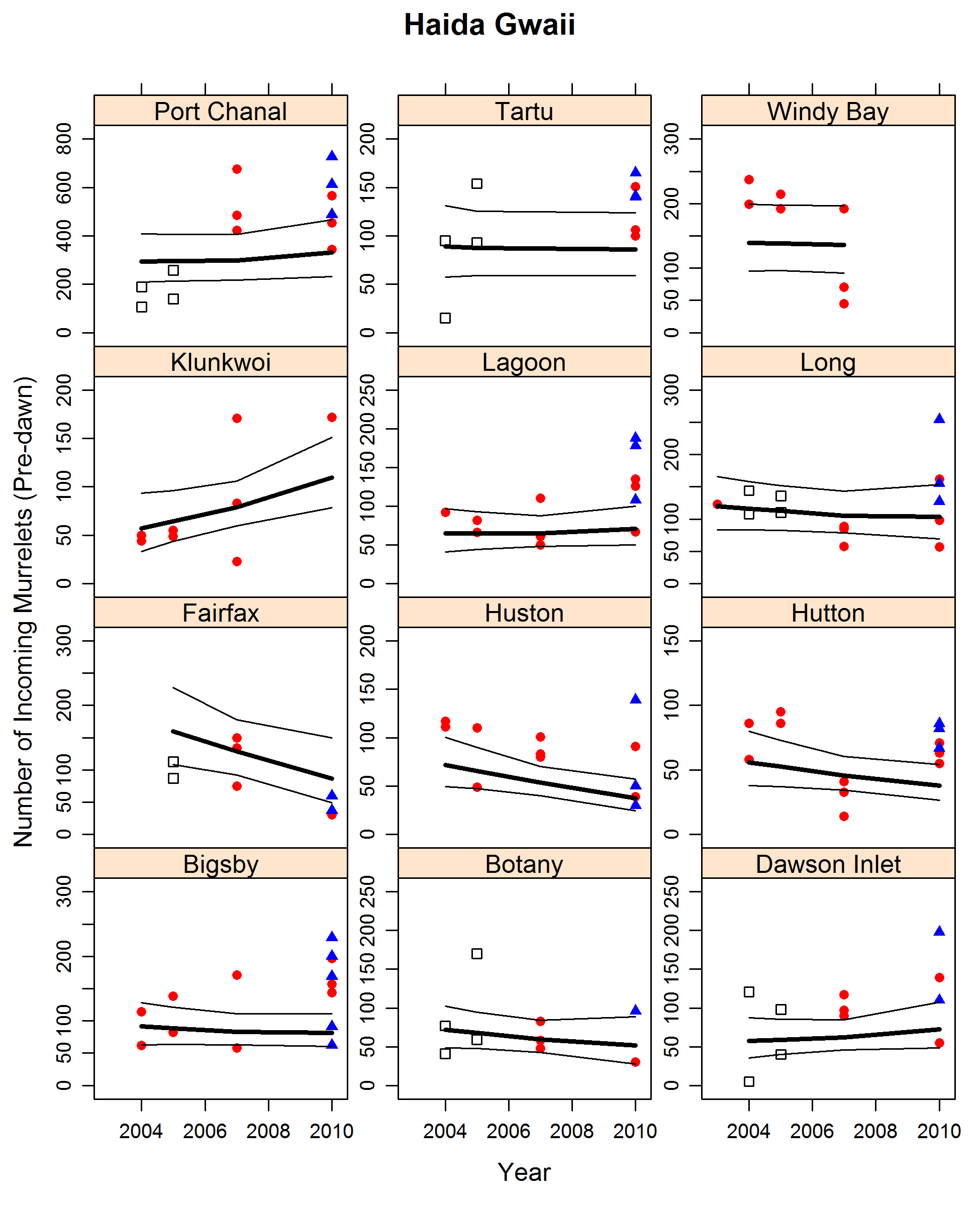

Supplement: S1 Fig — Lines are yearly estimates for each Region, as predicted by the trend model along with 95% credibility intervals. (TIF) [file pone.0134891.s005.tif]

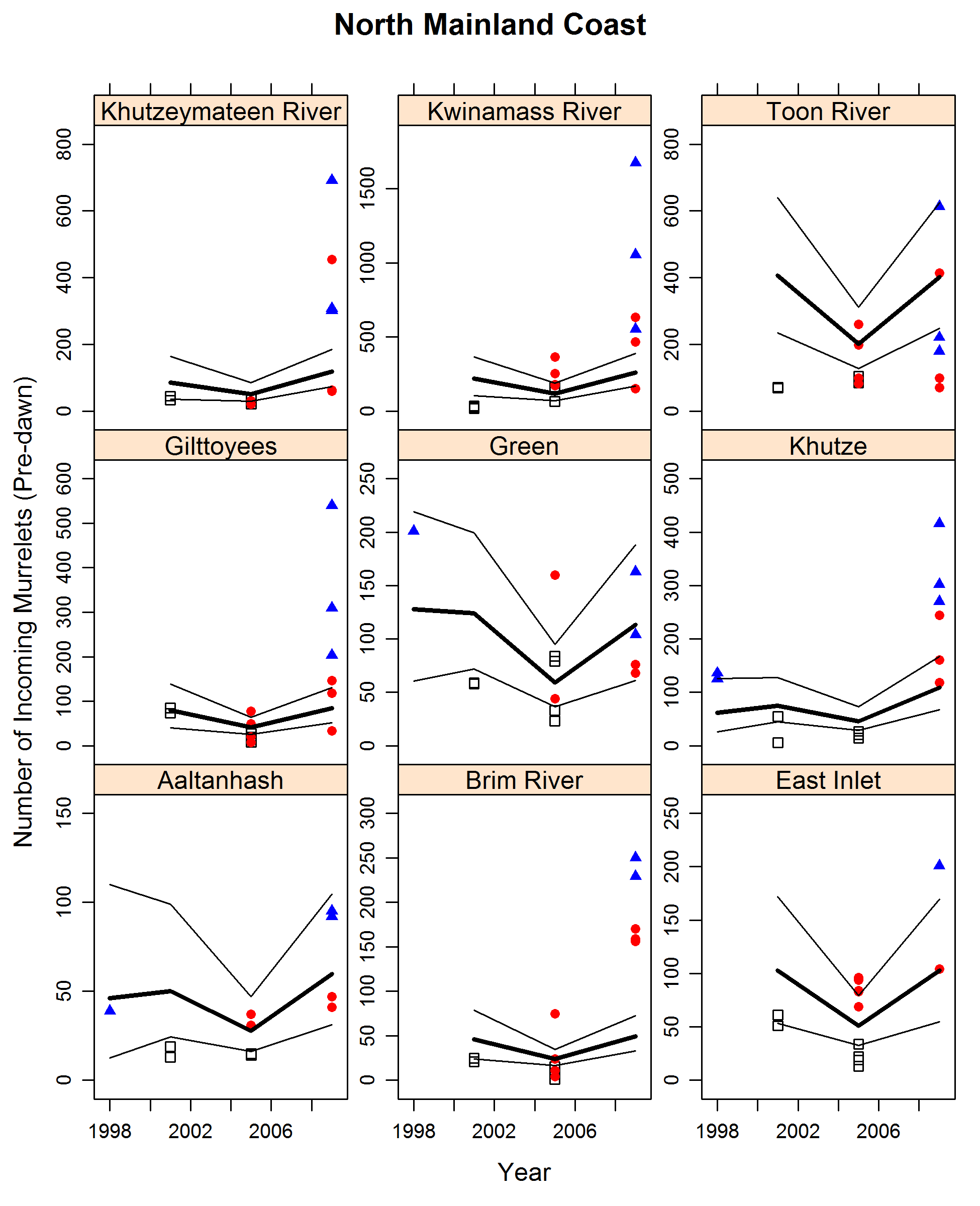

Supplement: S2 Fig — Lines are yearly estimates predicted by the trend model, along with 95% credibility intervals. (TIF) [file pone.0134891.s006.tif]

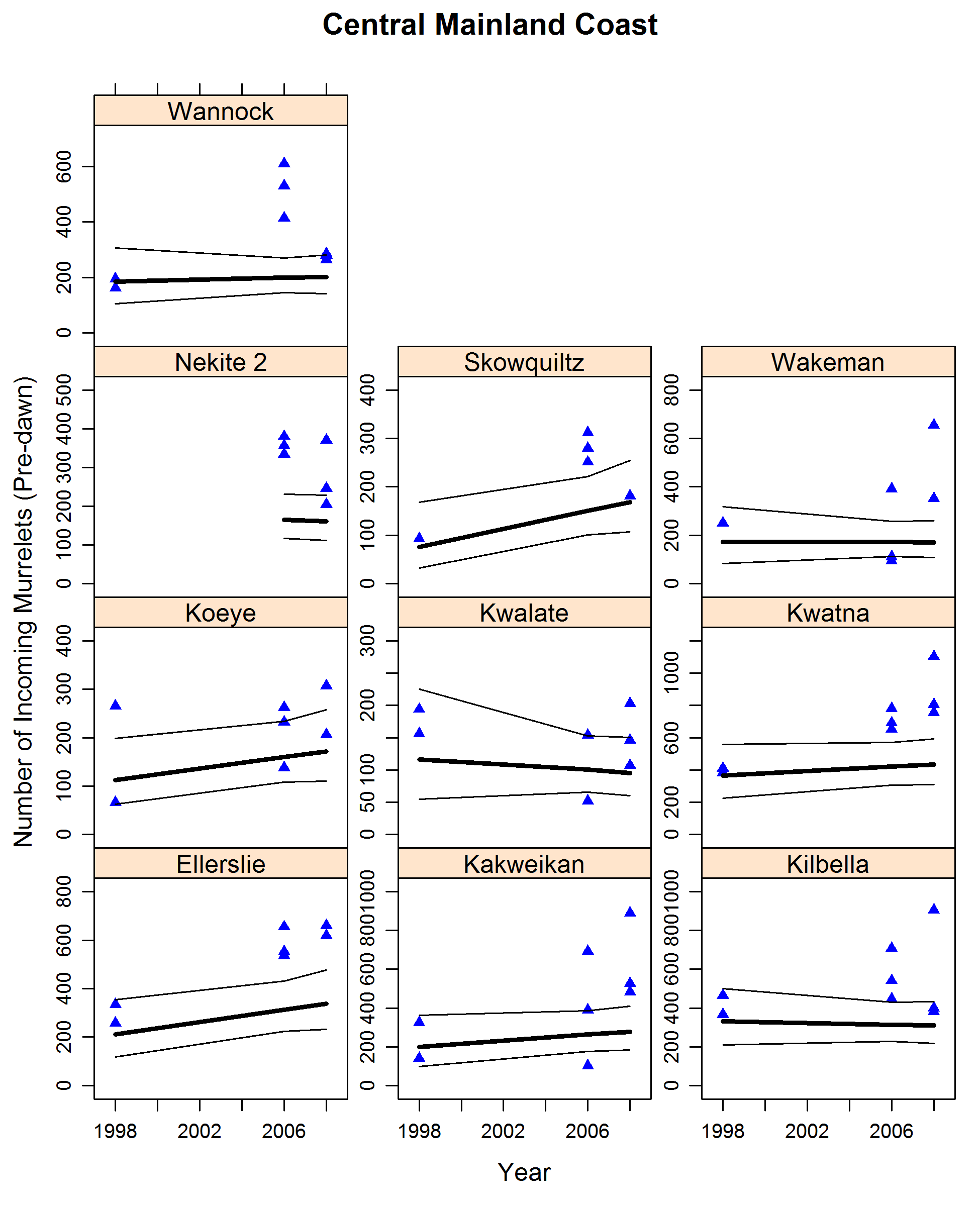

Supplement: S3 Fig — Lines are yearly estimates predicted by the trend model, along with 95% credibility intervals. (TIF) [file pone.0134891.s007.tif]

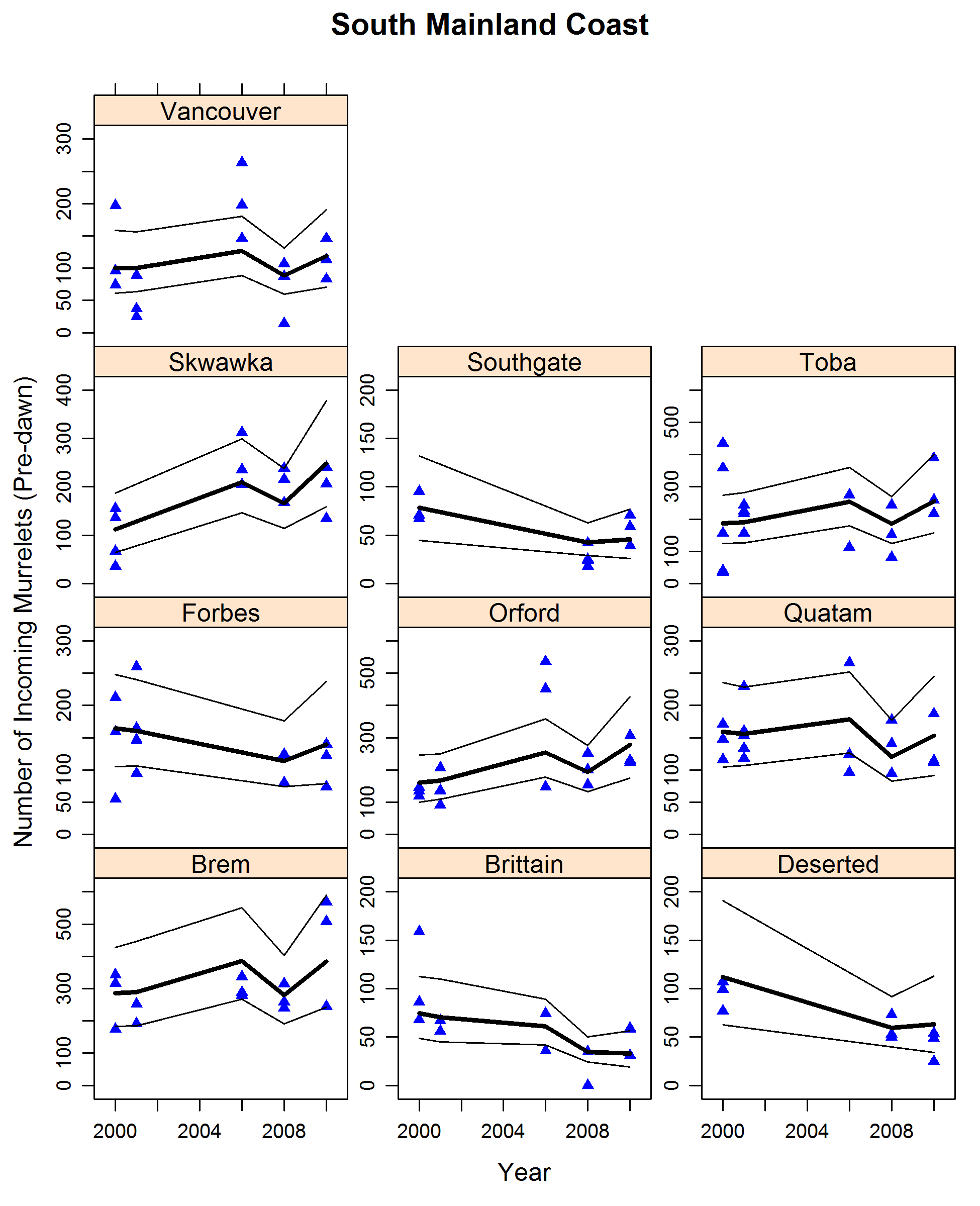

Supplement: S4 Fig — Lines are yearly estimates predicted by the trend model, along with 95% credibility intervals. (TIF) [file pone.0134891.s008.tif]

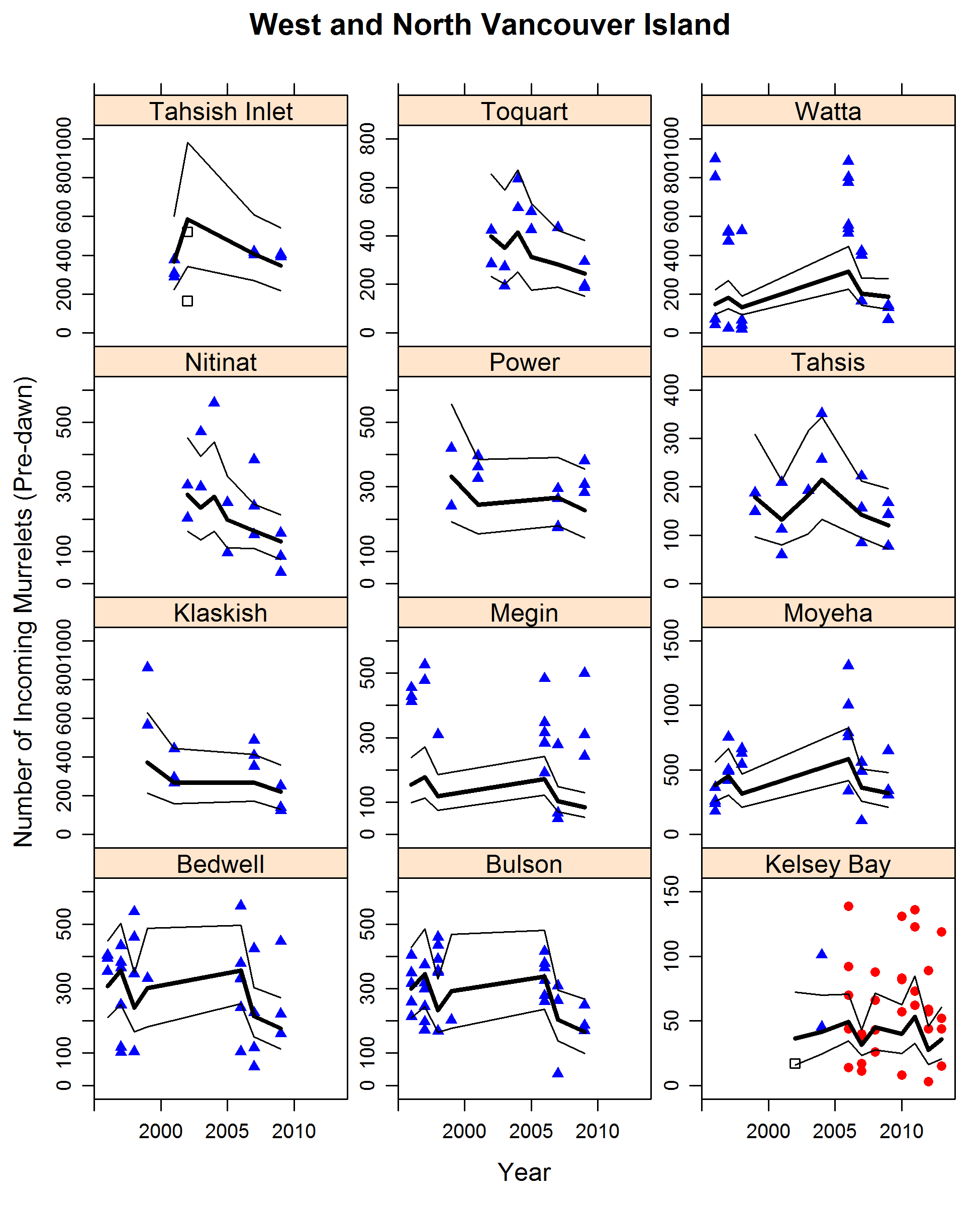

Supplement: S5 Fig — Lines are yearly estimates predicted by the trend model, along with 95% credibility intervals. (TIF) [file pone.0134891.s009.tif]

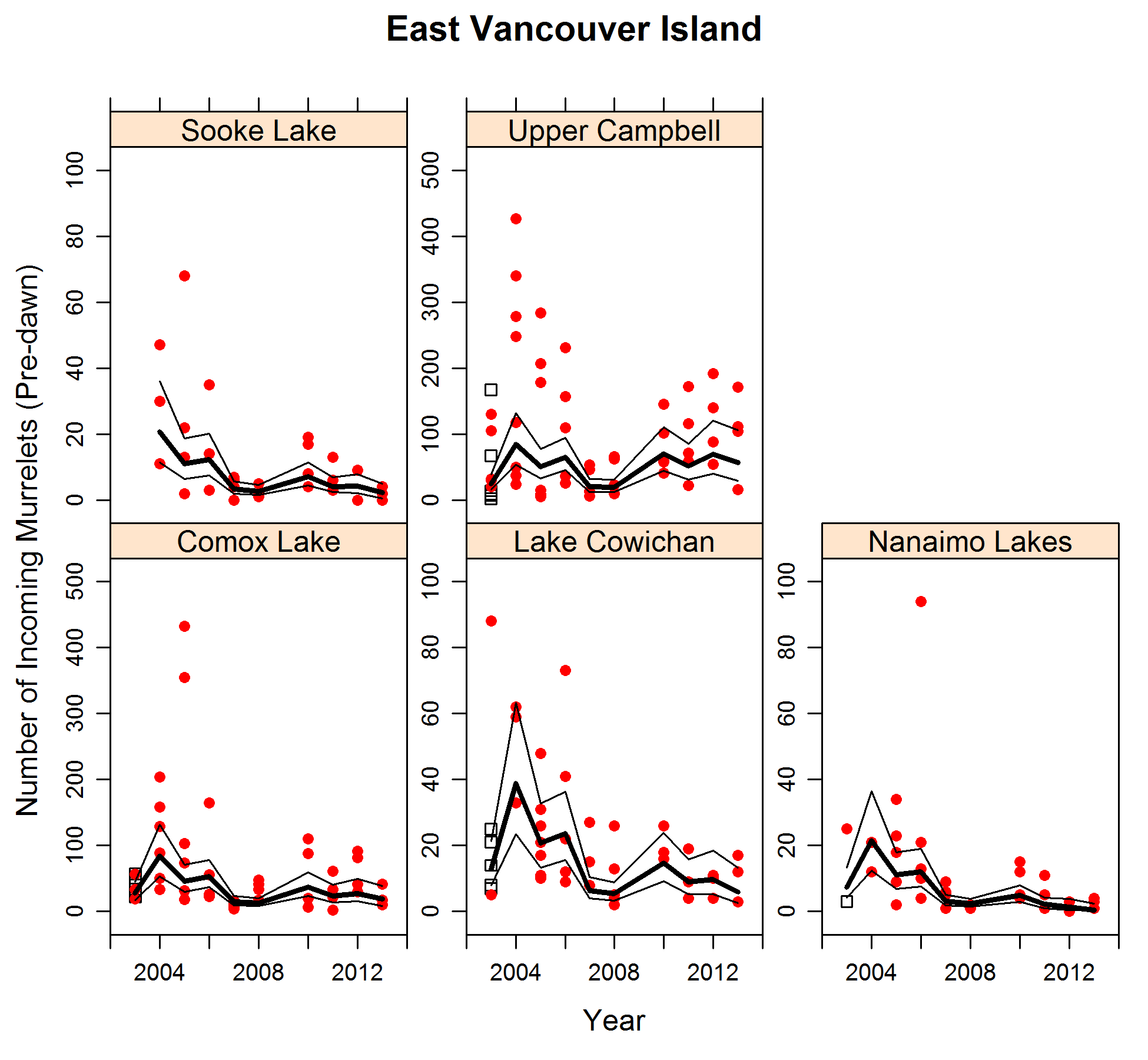

Supplement: S6 Fig — Lines are yearly estimates predicted by the trend model, along with 95% credibility intervals. (TIF) [file pone.0134891.s010.tif]
